# Estimates of gene ensemble noise highlight critical pathways and predict disease severity in H1N1, COVID-19 and mortality in sepsis patients

**DOI:** 10.1038/s41598-021-90192-9

**Published:** 2021-05-24

**Authors:** Tristan V. de Jong, Victor Guryev, Yuri M. Moshkin

**Affiliations:** 1 European Research Institute for the Biology of Ageing, University of Groningen, University Medical Centre Groningen, Groningen, The Netherlands; 2 Federal Research Centre, Institute of Cytology and Genetics, SB RAS, Novosibirsk, Russia; 3 Institute of Molecular and Cellular Biology, SB RAS, Novosibirsk, Russia; 4 Gene Learning Association, Geneva, Switzerland

**Keywords:** Bioinformatics, Gene expression analysis, Respiratory system models, Predictive markers, Prognostic markers

## Abstract

Finding novel biomarkers for human pathologies and predicting clinical outcomes for patients is challenging. This stems from the heterogeneous response of individuals to disease and is reflected in the inter-individual variability of gene expression responses that obscures differential gene expression analysis. Here, we developed an alternative approach that could be applied to dissect the disease-associated molecular changes. We define gene ensemble noise as a measure that represents a variance for a collection of genes encoding for either members of known biological pathways or subunits of annotated protein complexes and calculated within an individual. The gene ensemble noise allows for the holistic identification and interpretation of gene expression disbalance on the level of gene networks and systems. By comparing gene expression data from COVID-19, H1N1, and sepsis patients we identified common disturbances in a number of pathways and protein complexes relevant to the sepsis pathology. Among others, these include the mitochondrial respiratory chain complex I and peroxisomes. This suggests a Warburg effect and oxidative stress as common hallmarks of the immune host–pathogen response. Finally, we showed that gene ensemble noise could successfully be applied for the prediction of clinical outcome namely, the mortality of patients. Thus, we conclude that gene ensemble noise represents a promising approach for the investigation of molecular mechanisms of pathology through a prism of alterations in the coherent expression of gene circuits.

## Introduction

Both viral and bacterial pneumonia may lead to a life-threatening condition known as sepsis. The most notable cases in the public perception include pandemic viral infections such as the 2009 swine flu pandemic caused by H1N1^1^ and more recently, the 2019 coronavirus disease (COVID-19) caused by severe acute respiratory syndrome coronavirus 2 (SARS-CoV-2)^2^. Like with any other seasonal severe acute respiratory infections (SARI), these pandemics resulted in a significant rise in patients with sepsis in intensive care units^3,4^. Sepsis is a complex reaction of the host (human) to a systemic infection, viral or bacterial, often resulting in septic shock or death^5–7^. A problem of both sepsis treatment and the prediction of patients’ clinical outcomes relate to the highly heterogeneous nature of sepsis^8^. Despite recent progress in the identification of molecular biomarkers for sepsis^8–15^, treatment remains mainly non-curative and clinical outcomes are mostly inferred from clinical signs^6^.

A canonical approach for the identification of disease biomarkers and their potential therapeutic targets relies on differential gene expression (DGE) analysis either on RNA or protein levels. This stems from a classical gene regulation Jacob-Monod model, which implies a specific gene expression response (up- or down-regulation) to a specific signal (see the recent perspective on historical origins of the model^16^). However, gene expression is a stochastic process and cellular responses to signals often trigger a cascade of changes in gene expression, making it difficult to discover specific targets and biomarkers for disease.

The stochastic nature of gene expression implies a natural variation in RNA and protein copy numbers^17^. According to the fluctuation-response relationship^18,19^, an amount of gene expression response to a signal (fluctuation) is proportional to its variance (or the squared biological coefficient of variation—*bcv*^*2*^) for log-scaled values of RNA copy number^20^. Consequently, statistical inference of differentially expressed genes will be biased towards genes with high variance (*bcv*^*2*^) (Figure S1). This leads to a set of intrinsic problems with DGE analysis. (1) Genes with increased variability in expression will strongly respond to any cellular signal aimed at them, though these genes may not necessarily be causative for a diseased state. Even under normal circumstances, these exhibit large fluctuations and thus are loosely-regulated. (2) In contrast, genes with a low variability will respond only modestly. These genes are tightly-regulated and any fluctuations in their expression might be causative for a diseased state.

Upon calling significantly changed genes, to make biological sense, these genes are mapped to known biological pathways such as GO or KEGG^21,22^, or subunits of protein complexes annotated by CORUM or other interaction databases^23^. Thus, a second statistical test is required, namely gene set enrichment analysis (GSEA). However, this is not without its own caveats. The major one is that GSEA depends on the statistical inference of DGE and DGE cut-offs^24,25^. As a result, biological interpretations from DGE might be drastically affected by pitfalls arising from the fluctuation-response relationship, DGE thresholding, and the choice of a statistical approach for GSEA.

To circumvent this, we reasoned that (1) genes do not function in isolation, but rather act as ensembles representing biological pathways and/or subunits of protein complexes. (2) The normal function of a biological pathway or protein complex requires a regulated (balanced) expression of the whole gene ensemble. (3) Any alterations in the expression of a gene ensemble might be causative for a disease or predictive for the clinical outcome. To infer the alterations in gene ensembles expression we turned to the estimation of their variances (gene ensemble noise) from whole blood gene expression profiles of individuals under normal and pathological conditions. From the total law of variance, gene ensemble noise (${\text{Var}}\left[ G \right]$) sums from the variance of the ensemble genes’ means and (${\text{Var}}\left[ {{\text{E}}\left[ {G|g} \right]} \right]$), as well as the expectation of ensemble genes variances (${\text{E}}\left[ {{\text{Var}}\left[ {G|g} \right]} \right]$) (Figure S2). Thus, the gene ensemble noise estimates both: changes in stoichiometries of genes encoding either a biological pathway or protein complex subunits, and changes in mean gene expression variability for genes in the ensemble.

From the whole blood expression profiles of patients under intensive care treatment, we estimated how gene ensemble noise corresponds to a pathological state, such as sepsis, e.g. community/hospital-acquired pneumonia (CAP), viral H1N1 pneumonia (H1N1) and COVID-19 disease. From this analysis, we identified a number of pathways for which gene ensemble noise associated positively with an individual health/disease state or mortality risk when treated as an ordinal variable (healthy < early H1N1 phase < late H1N1 phase, healthy < sepsis (CAP, other) survived < sepsis (CAP, other) deceased patients, and less-severe < severe COVID-19). Finally, we identified pathways and complexes where deregulation is associated with sepsis mortality risk (CAP, other) and severity of the COVID-19 disease based on gene ensemble noise with high accuracy. Our findings were also corroborated with the weighted correlation network analysis (WGCNA). We concluded that the gene ensemble noise provides a powerful tool for the discovery of systemic disease biomarkers, pharmaceutically targetable pathways, and the prediction of a disease clinical outcome.

## Results

### Mean and variance gene expression response to infection and sepsis

Sepsis is thought to trigger a plethora of heterogenous host responses to a systemic infection^6,8^. We reasoned that this heterogeneity might be reflected in the inter-individual gene expression variability (standard deviation—σ or variance—σ^2^). Considering that (a) The RNA copy number is a mixed Poisson (*e.g.* negative binomial) random variable^26^ and that (b) log-transformed microarray hybridization signal intensities correlate with log-transformed RNA-seq copy numbers^27^. It is easy to show that the variance of log gene expression approximates the biological coefficient of variation (bcv^2^)^20^. From the first-order Taylor expansion for variance: ${\upsigma }_{Y}^{2} \approx \frac{{{\upsigma }_{X}^{2} }}{{\overline{X}^{2} }} = cv_{X}^{2}$, where $Y = log\left( X \right)$ is the log gene expression. The mixed Poisson random variable, $cv_{X}^{2} = \frac{1}{{\mu_{X} }} + bcv^{2}$, where *bcv*^*2*^, also known as the overdispersion parameter, is independent of mean gene expression ($\mu_{X}$). Thus, for $\mu_{X} \gg 1$ (for genes with a large mean RNA copy number), ${\upsigma }_{Y}^{2} \approx bcv^{2}$. In other words, by estimating the inter-individual log gene expression variabilities, from either microarray signal intensities or RNA-seq counts, we can infer approximately the biological coefficients of variations from genes’ RNA copy numbers.

**Figure 1:**
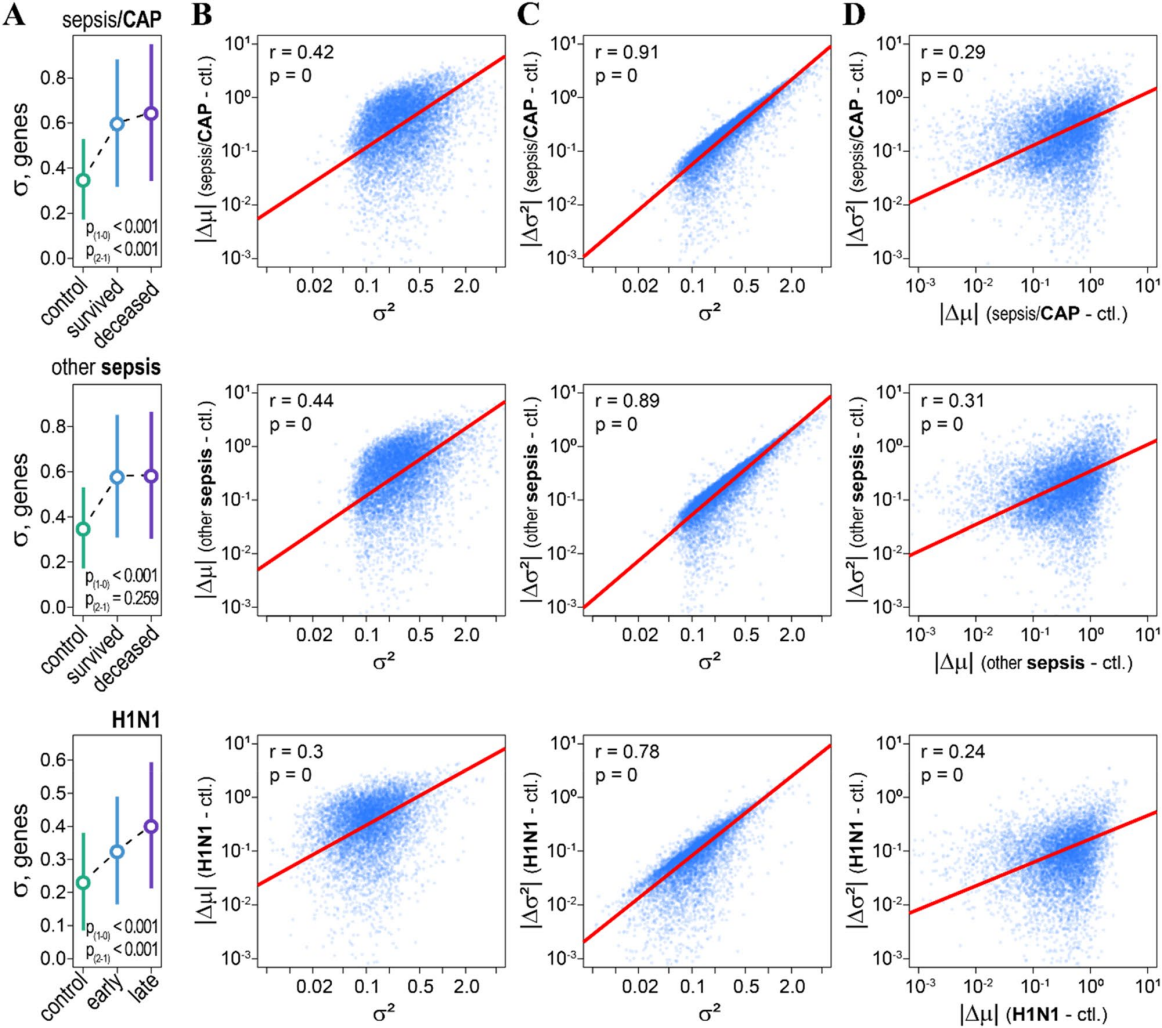
H1N1 and sepsis coordinately affect mean gene expression and inter-individual gene expression variability. (**A**) Inter-individual variability in whole blood gene expression (σ) increases in sepsis/CAP (top), other sepsis (mid), and H1N1 (bottom) patients as compared to healthy individuals. p_(1–0)—_*p*-values of *t-*tests comparing differences in inter-individual gene expression variability of healthy individuals (control) with survived (sepsis) and early H1N1 infected patients. p_(2–1)—_*p*-values of *t-*tests comparing differences of survived (sepsis) and early H1N1 infected patients with deceased (sepsis) and late H1N1 infected patients. Circles and whiskers indicate means and standard deviations respectively. (**B**) Correlations between variances in whole blood gene expression (σ^2^) and absolute changes in mean gene expression (|Δμ|) for healthy individuals (ctl.) and patients (sepsis, H1N1). Due to the fluctuation-response relationship, the magnitude of the mean gene expression response depends on its variance. We estimated common variances for genes in healthy and sepsis/CAP patients (top), healthy and other sepsis patients (mid), and healthy and H1N1 patients (bottom). (**C**) Correlations between variances in whole blood gene expression (σ^2^) and absolute changes in inter-individual gene expression variability (|Δσ^2^|) for control individuals (ctl.) and patients (sepsis, H1N1). (**D**) Correlations between absolute changes in mean gene expression (|Δμ|) and in inter-individual gene expression variability (|Δσ^2^|).

We estimated the dispersions for whole blood log gene expressions in sepsis patients (8826 genes), and H1N1 infected patients (7240 genes) from the two data sets GSE65682 and GSE21802 respectively (for a detailed description of cohorts see original studies and Methods)^8,12,28^. For sepsis patients we also accounted for age, including it as a random variable in the Generalized Additive Model for Location, Scale and Shape (GAMLSS)^29^, see “Methods” section. On average, the dispersions in log gene expressions in sepsis, and H1N1 patients were significantly higher as compared to healthy individuals (Fig. 1A). To that, for sepsis/CAP patents’ dispersions in log gene expressions were significantly higher for deceased patients as compared to those who survived. Likewise, for H1N1 patients, dispersions in log gene expressions further increased in the late phase of infection (Fig. 1A). For sepsis patients, on average, dispersions of log gene expressions were comparable between survived and deceased patients for all analysed genes (Fig. 1A). However, for genes for which the dispersions changed significantly between healthy individuals and sepsis patients (Bonferroni adjusted *p* ≤ 0.05), their dispersions on average were higher among the deceased patients as compared to those that survived (*p* < 0.001). Together, these suggest that the host response to infection increases the biological coefficients of variations in genes’ RNA copy numbers (as ${\upsigma }^{2} \approx bcv^{2}$) and substantiates heterogeneity in the pathogenesis of sepsis^8^ from the gene expression perspective.

Because of the fluctuation-response relationship^18^, absolute changes in the mean log gene expressions (|Δμ|) in response to infection (sepsis/CAP, H1N1) and sepsis correlated significantly with the variances of the log gene expressions (Fig. 1B). Interestingly, we also noted significant correlations between the absolute changes in inter-individual gene expression variabilities (|Δσ^2^|) and the variances of log gene expressions (Fig. 1C). Consequently, |Δμ| and |Δσ^2^| were also correlated (Fig. 1D). From this, we conclude that H1N1 and sepsis result in coordinated changes in both the mean and the heterogeneity of the expression of genes and that magnitudes of these changes depend on the genes’ biological coefficients of variation.

### Gene ensemble noise response to infection and sepsis

Both the mean and variance relate to population (inter-individual) statistics reflecting distinct aspects of gene regulation. Changes in means fit the classical DGE view on gene response to pathology and other biological processes, while changes in variances yield a view on the heterogeneity of the gene response. However, as noted before (Fig. 1 and S1), statistical inference of these changes is biased towards higher significance for genes with a high biological coefficient of variation. Although changes in RNA copy number can serve in practical applications for diagnostics of disease and clinical outcomes, inter-individual variability cannot be used for diagnosis. At the same time, stochastic fluctuations in gene expression remain attractive for the dissection of novel molecular mechanisms of pathology. Therefore, we expect that the estimation of gene ensemble noise may provide additional benefits for diagnostics by quantifying fluctuations while being informative for personalized treatment.

We define gene ensemble noise as the variance of log-transformed, normalized expression levels for a collection of genes $G = \left( {g_{1} , \ldots ,g_{i} } \right)$ encoding for either a protein of a pathway or subunits of a protein complex. To this end, we mapped genes to the KEGG-annotated pathways and the CORUM-annotated protein complexes^22,23^. From the law of total variance: ${\text{Var}}\left[ G \right] = {\text{E}}\left[ {{\text{Var}}\left[ {G|g} \right]} \right] + {\text{Var}}\left[ {{\text{E}}\left[ {G|g} \right]} \right]$, gene ensemble noise depends on the variability in expression of genes in ensemble (${\text{E}}\left[ {{\text{Var}}\left[ {G|g} \right]} \right]$) and on their stoichiometry (${\text{Var}}\left[ {{\text{E}}\left[ {G|g} \right]} \right]$) (Figure S1). Thus, the simple estimation of the variances (${\text{Var}}\left[ G \right]$) of gene ensembles for each individual might reflect alterations in the function of biological pathways and protein complexes on the level of stoichiometry and gene noise.

**Figure 2:**
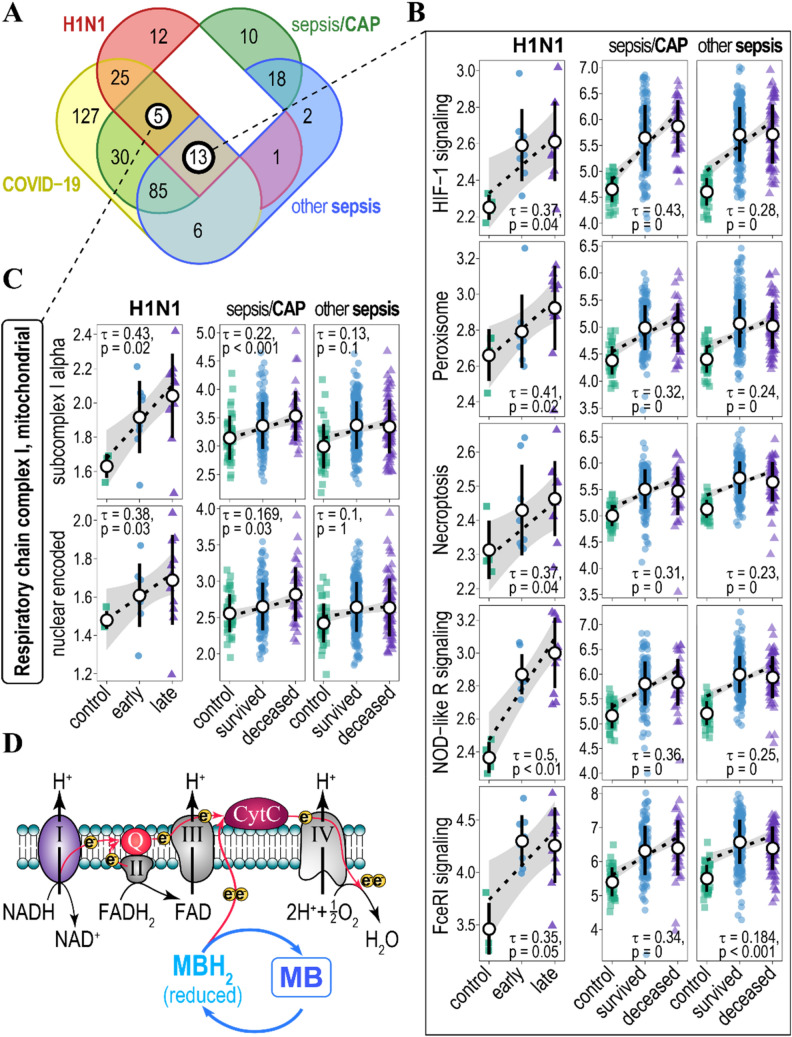
Association of gene ensemble noise with H1N1 infection phase and sepsis mortality. (**A**) Venn diagram of KEGG- and CORUM-annotated biological pathways/protein complexes for which gene ensemble noise associates positively (increases) and significantly with disease state (mild/severe for the COVID-19, healthy/early/late for the H1N1, and healthy/survived/deceased for sepsis/CAP and other sepsis patients). (**B**) Plots of gene ensemble noise for genes involved in HIF-1 signalling, peroxisome, necroptosis, NOD-like receptor, and Fc epsilon RI signalling pathways. Pathways were annotated by KEGG. Kendall tau, and FDR- (H1N1 patients) and Bonferroni- (sepsis patients) adjusted *p*-values are indicated. Rank-based regression trend lines and 95% confidence bands of gene ensemble noise association with the state of disease are shown. Black circles and whiskers indicate means and standard deviations. (**C**) Plots of gene ensemble noise for genes encoding CORUM-annotated subunits of mitochondrial respiratory chain complex I (subcomplex I alpha—top panel and nuclear-encoded subunits—bottom panel). Rank-based regression trend lines and 95% confidence bands of gene ensemble noise association with the state of the disease are shown. (**D**) Methylene Blue (MB) acts as an alternative electron donor to the electron transport chain (red arrows) by shuttling between redox states (MB—MBH_2_) and, thus, bypassing respiratory chain complex I. Respiratory chain complex I-IV and their substrates are indicated, Q—coenzyme Q10, CytC—cytochrome C. Electrons are indicated as yellow circles.

We correlated ${\text{Var}}\left[ G \right]$ for ensembles with H1N1 and sepsis disease states. For H1N1 viral infection, the disease state can clearly be ranked: non-infected (healthy) < early phase < late phase of infection, thus it represents an ordinal variable^28^. For sepsis patients, we assumed that a condition of the deceased patients was worse than that of those who survived. We considered that healthy < survived < deceased can also be represented as an ordinal disease state variable. Circumstantially, this is supported by distinct blood gene expression endotypes^8^ and an increased gene expression heterogeneity (Fig. 1A). Kendall rank correlation identified a number of pathways and protein complexes for which the gene ensemble noise was positively and significantly associated with the disease state in H1N1 (FDR ≤ 0.05) and sepsis patients (Bonferroni-adjusted *p* ≤ 0.05) (Fig. 2A). None of the pathways or gene complexes were negatively associated with the disease state at the specified significance thresholds. We used different *p*-value adjustment procedures (FDR—less conservative, and Bonferroni—more conservative) for H1N1 and sepsis patients due to the large differences in sample sizes (number of patients) between these data sets.

Out of all gene ensembles, 13 of them proved to be consistent and correlated to the increased disease state in gene ensemble noise in all three disease conditions (Fig. 2A,B, Table S1A). Most of these gene ensembles (pathways) are known to be involved in the pathology of sepsis through multiple pieces of experimental evidence (Table 1), thus substantiating the power of gene ensemble noise analysis. However, gene ensemble noise yields novel insights into the molecular mechanisms of sepsis (H1N1, CAP, or other-causes of sepsis) by suggesting a holistic misregulation in stoichiometry and gene noise for these gene ensembles.

**Table 1:** Role in sepsis of the pathways for which gene ensemble noise associates positively (increases) with the disease states (healthy < early/survived < late/deceased).

Pathways/Complexes	Role in sepsis pathology	References
KEGG: HIF-1 signalling pathway	Metabolic reprogramming of innate immune cells during the hyperinflammatory and immunotolerant phases of sepsis	^30,31^
KEGG: Peroxisome	Defective peroxisome recycling alters cellular redox homeostasis and leads to exaggerated oxidative stress response to endotoxin (infection) and sepsis	^32^
KEGG: Necroptosis	Necroptosis is implicated in pulmonary diseases and sepsis-associated organ injury	^33,34^
KEGG: NOD-like receptor signalling pathway	Activation of Toll-like and NOD-like receptor signalling protects mice from polymicrobial sepsis-associated lethality	^35^
KEGG: Fc epsilon RI signalling pathway	Fc receptors bind to antibodies attached to invading pathogens and their up-regulation can serve a potential biomarker for sepsis. Mice deficient for FCER1G gene encoding the γ-subunit of Fc epsilon RI show increased resistance to sepsis	^36,37^
KEGG: Autophagy—other	Autophagy is an adaptive protective process that eliminates damaged proteins, organelles and pathogens. It is thought to be a promising target in treatment of sepsis	^38^
KEGG: Biosynthesis of amino acids	Sepsis results in significant disorders in amino acids metabolism	^39^
KEGG: Glucagon signalling pathway	Glucagon levels negatively associate with clinical outcome in sepsis patients	^40^
KEGG: Propanoate (propionate) metabolism	Propionic acidaemia caused by altered propionate metabolism often results in sepsis and death	^41^
KEGG: Circadian rhythm	There is accumulating evidence for association between circadian misalignment and severity of inflammatory responses in sepsis	^42^
KEGG: Dopaminergic synapse	Dopamine mediates neuroimmune communications and dopaminergic is implicated in inflammation and sepsis	^43,44^
KEGG: Amyotrophic lateral sclerosis (ALS)	ALS patients often develop pulmonary insufficiency and have increased risk of sepsis	^45^
CORUM: Respiratory chain complex I, mitochondrial	Mitochondrial disfunction resulting in reduced respiratory chain complex I activity and low ATP levels is a whole mark for sepsis	^46^
KEGG: Osteoclast differentiation	Mean expression of osteoclast differentiation genes is up-regulated in human septic shock	^47^
KEGG: Tight junction	Sepsis disrupts intestinal barrier which leads to a multiple organ dysfunction syndrome and alters the expression of tight junction proteins	^48^

We also identified 5 gene ensembles for which gene ensemble noise was positively and significantly correlated with the disease state in H1N1 and CAP/sepsis patients (Fig. 2A, Table S1B). However, gene ensemble noise for these pathways was also significantly increased in sepsis patients (t-test, Bonferroni adjusted *p* < 0.01) despite insignificant rank correlation. To that, some of these pathways can be implicated in the pathology of sepsis (Table 1). Two of these ensembles were represented by genes encoding mitochondrial respiratory chain complex I (Complex I) (Fig. 2C). From the point of view of gene ensemble noise, this suggests an altered stoichiometry and gene noise in the expression of the subunits of the Complex I which, as a result, might lead to its improper assembly and function in H1N1 and sepsis patients. It has previously been established that the activity of the Complex I is decreased and correlates negatively with the sepsis disease outcome^46^. Complex I is the first set of enzymes of the respiratory chain and it is the entry point for most electrons into the electron transport chain^49^. Interestingly, in the case of Complex I inhibition or deregulation, methylene blue (MB) can bypass it by acting as an alternative redox mediator in the electron transport chain, thus restoring mitochondrial respiration^50,51^ (Fig. 2D). MB is also considered to be a promising therapeutic in the treatment of septic shock^52,53^. Thus, gene ensemble noise might provide a simple yet powerful explanatory shortcut from the expression of thousands of genes, to the function of gene ensembles leading to possible pharmaceutical targets.

### Predicting clinical outcome for sepsis patients from the gene ensemble noise

Treatment of sepsis is challenging and mortality rates among sepsis patients are high. Yet, prediction of clinical outcome is also challenging due to the heterogeneity in both the pathology^8^ and gene expression (Fig. 1A). Recently, the Molecular Diagnosis and Risk Stratification of Sepsis (MARS) consortium identified the Mars1 gene expression endotype which was significantly associated with acute (28-day) mortality, however, for other endotypes, Mars2-4 poorly discriminated between the survival and mortality of patients^8^. Thus, we wondered whether the clinical outcome (mortality) could be predicted from the gene ensemble noise.

To this end, we trained binary logistic gradient boosted regression tree models using survival and acute mortality as a binary response variable for clinical outcome and the patients’ age and blood gene ensemble noise as model features. The models were trained with XGBoost^54^. The sepsis patients were split into discovery (263 patients: 105 sepsis/CAP and 158 sepsis patients) and validation (216 patients: 78 sepsis/CAP and 138 sepsis patients) cohorts following GSE65682 annotation^8^. The discovery cohort was represented by the patients admitted for sepsis (including non-CAP patients) to intensive care units (ICUs) in the Netherlands between 2011 and 2012^12^, and the validation—by the patients with sepsis due to CAP admitted to ICUs in the UK between 2006 and 2014^55^. Within the cohorts, the mortality rates were 26.2% for sepsis patients (23.8% for sepsis/CAP and 27.8% for other sepsis patients) within the discovery cohort, and 20.8% for sepsis patients (19.2% for sepsis/CAP and 21.7% for other sepsis patients) in the validation cohort.

Overall, class-imbalance, noise due to the inter-individual heterogeneity, and high-dimensionality of model features are among the major problems of machine learning^56^. In part, gene ensemble noise leads to a reduction in inter-individual variability (Figure S3) and in dimensionality as model features are represented not by individual genes, but by collections of genes. Nonetheless, we further reduced the number of gene ensemble noise features in models by t-test feature selection. For this, we compared gene ensemble noise between survived and deceased patients in the discovery cohorts. The *p*-value cut-offs for the model features were selected based on the maximization of models’ training accuracy (see “Methods” section). XGBoost hyper-tuning parameters: learning rate, complexity, depth, etc*.* were optimized based on the cross-validation. To avoid overfitting, we used early epoch stopping, which was estimated from the test fold of the discovery cohort (see “Methods” section). Because of the class-imbalance, AUC (area under the receiver operating characteristic (ROC) curves) were used to evaluate the model performance. The validation cohorts were hidden from the feature selection and training.

**Figure 3:**
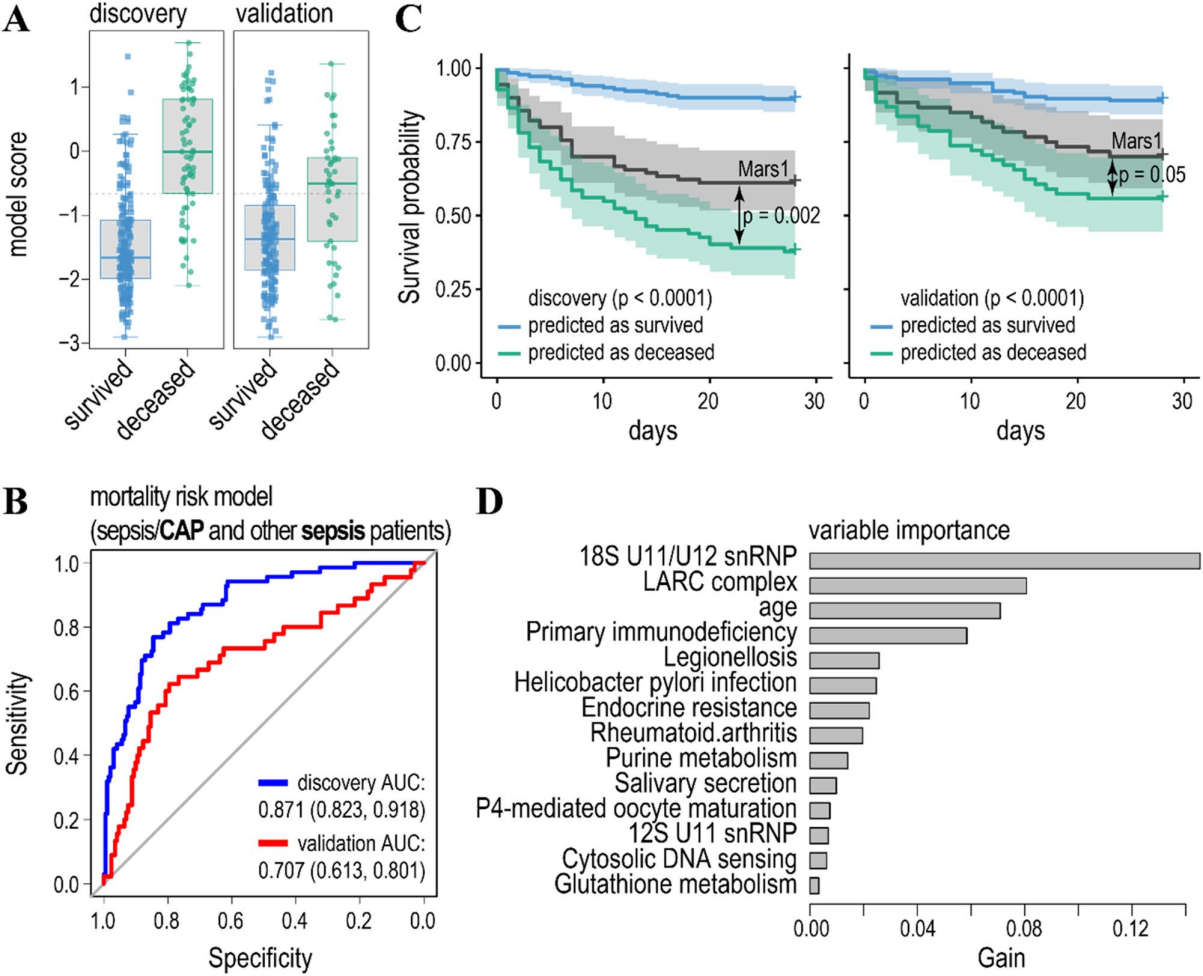
The model predicting mortality/survival of sepsis (grouped as sepsis/CAP and other sepsis) patients. (**A**) Boxplots of the model scores predicting mortality/survivorship in the discovery (left) and validation (right) cohorts. The model was trained on the same data as the published discovery cohort by the gradient boosted regression tree and validated on an independent cohort^8^. Dashed lines indicate threshold levels of classification. The threshold was calculated by maximizing a product of the specificity and sensitivity of the model prediction in the discovery cohort. Further details of model accuracy are given in Tables 2 and S2. (**B**) Receiver operating characteristic curves (ROC) for the model predicting mortality (endpoint—survival or death within 28 days after treatment) in sepsis (grouped as sepsis/CAP and other sepsis) patients (blue line—discovery cohort, red line—validation cohort). Features were selected by the t-test comparing gene ensemble noise between the survived and deceased patients in the discovery cohort to achieve maximum prediction accuracy for the discovery cohort. Values for the area under the ROC curve (AUC) are indicated. (**C**) Survival probability for the patients predicted to have low (blue line) and high (green line) risk of mortality for the discovery (left panel) and validation (right panel) cohorts. *P*-values indicate significant differences in hazards for the predicted classes (survival/mortality) according to the Cox proportional-hazards model. Black lines—survival probability of patients with Mars1 endotype^8^ was compared with the predicted deceased class for the discovery and validation cohorts. (**D**) Variable importance of the model ranks gene ensemble noise features according to their relative contribution (gain).

Figure 3A,B show model scores and ROC curves for the model, predicting mortality/survival for the sepsis patients in the discovery and validation cohorts. AUCs for the discovery and validation cohorts were 0.871 and 0.707 respectively, suggesting a reasonable accuracy of the model. However, from the model scores, and evaluation of the model specificity/sensitivity it appears that the model is biased towards the prediction of the major class (survived) (Fig. 3A, Table 2, and Table S2A). Thus, class prediction balanced accuracies (bACC = Specificity/2 + Sensitivity/2) were 0.799 and 0.701 for the discovery and validation cohorts respectively. Nonetheless, the survival probability for patients predicted to have a high risk of mortality was significantly lower than the survival probability for patients predicted to have a low risk of mortality in both the discovery and validation cohorts. To that, our model better predicts the risks of mortality as compared to the Mars1 endotype inferred from the log gene expression unsupervised learning^8^ (Fig. 3C). Potentially, this could be due to a lower inter-individual variability of gene ensemble noise as compared to log gene expression (Figure S3).

**Table 2:** Prediction accuracy of the models for the sepsis and COVID-19 patients based on the gene ensemble noise and WGCNA eigengenes explanatory variables for the discovery (Disc.) and validation (Valid.) cohorts.

Metric	Sepsis		Sepsis/CAP		Other sepsis		COVID-19	
	Disc	Valid	Disc	Valid	Disc	Valid	Disc	Valid
**Gene ensemble noise**								
bACC	0.799	0.701	0.802	0.798	0.779	0.761	0.963	0.95
Sensitivity	0.754	0.6	0.88	0.867	0.75	0.8	0.974	1
Specificity	0.845	0.801	0.725	0.73	0.807	0.722	0.951	0.9
**WGCNA eigengenes**								
bACC	0.805	0.707	0.8	0.806	0.82	0.734	0.963	0.9
Sensitivity	0.799	0.614	0.8	0.867	0.798	0.769	0.974	0.9
Specificity	0.812	0.8	0.8	0.746	0.841	0.7	0.951	0.9

**Figure 4:**
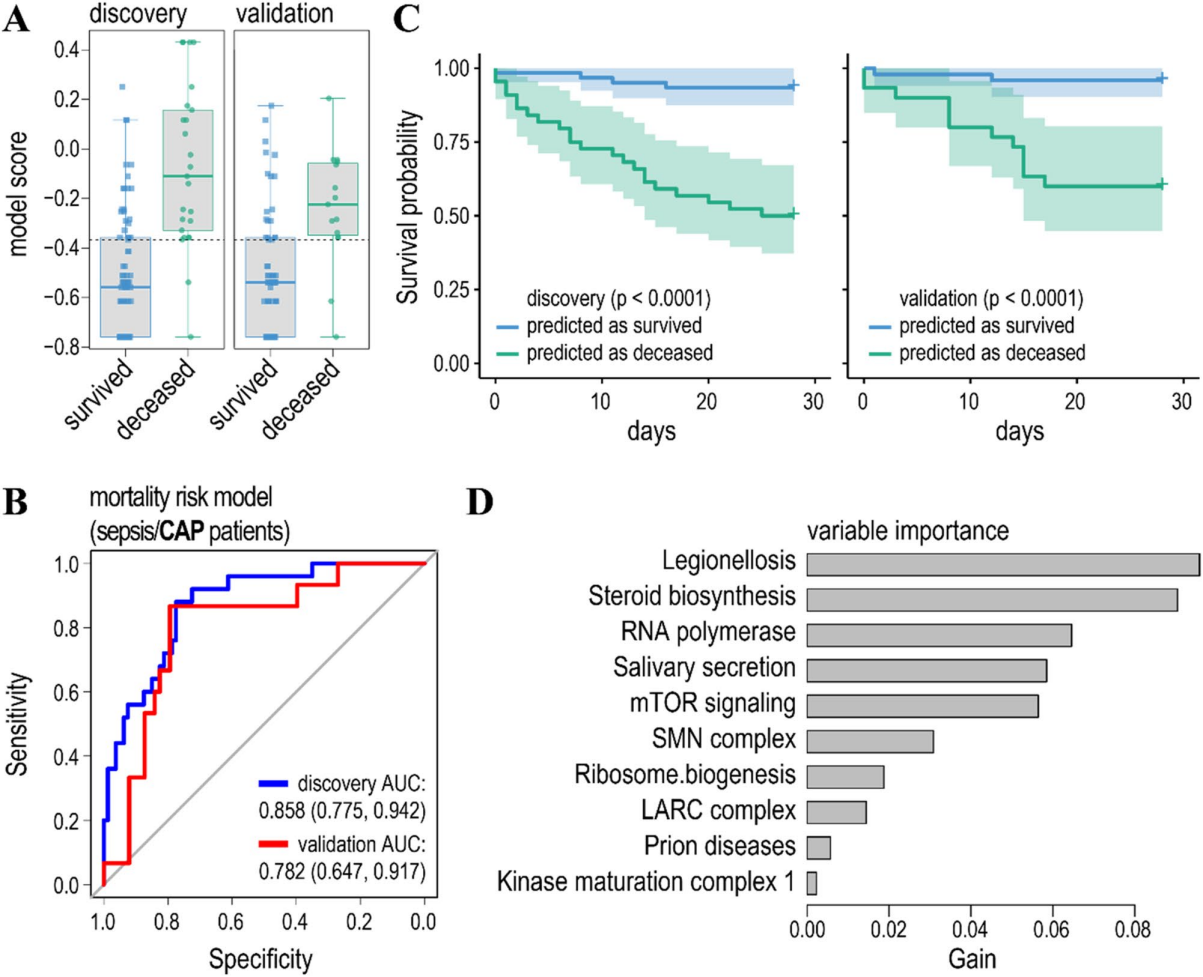
The model predicting mortality/survival of sepsis/CAP patients. (**A**) Boxplots of the model scores predicting mortality/survivorship in the discovery (left) and validation (right) cohorts. (**B**) ROC curves for the model predicting mortality in sepsis/CAP patients in the discovery (blue line) and validation (red line) cohorts. Cohorts were partitioned as in the previous study^8^. (**C**) Survival probability for the patients predicted to have low (blue line) and high (green line) risk of mortality for the discovery (left panel) and validation (right panel) cohorts. (**D**) Relative contribution of gene ensemble noise features to the model.

**Figure 5:**
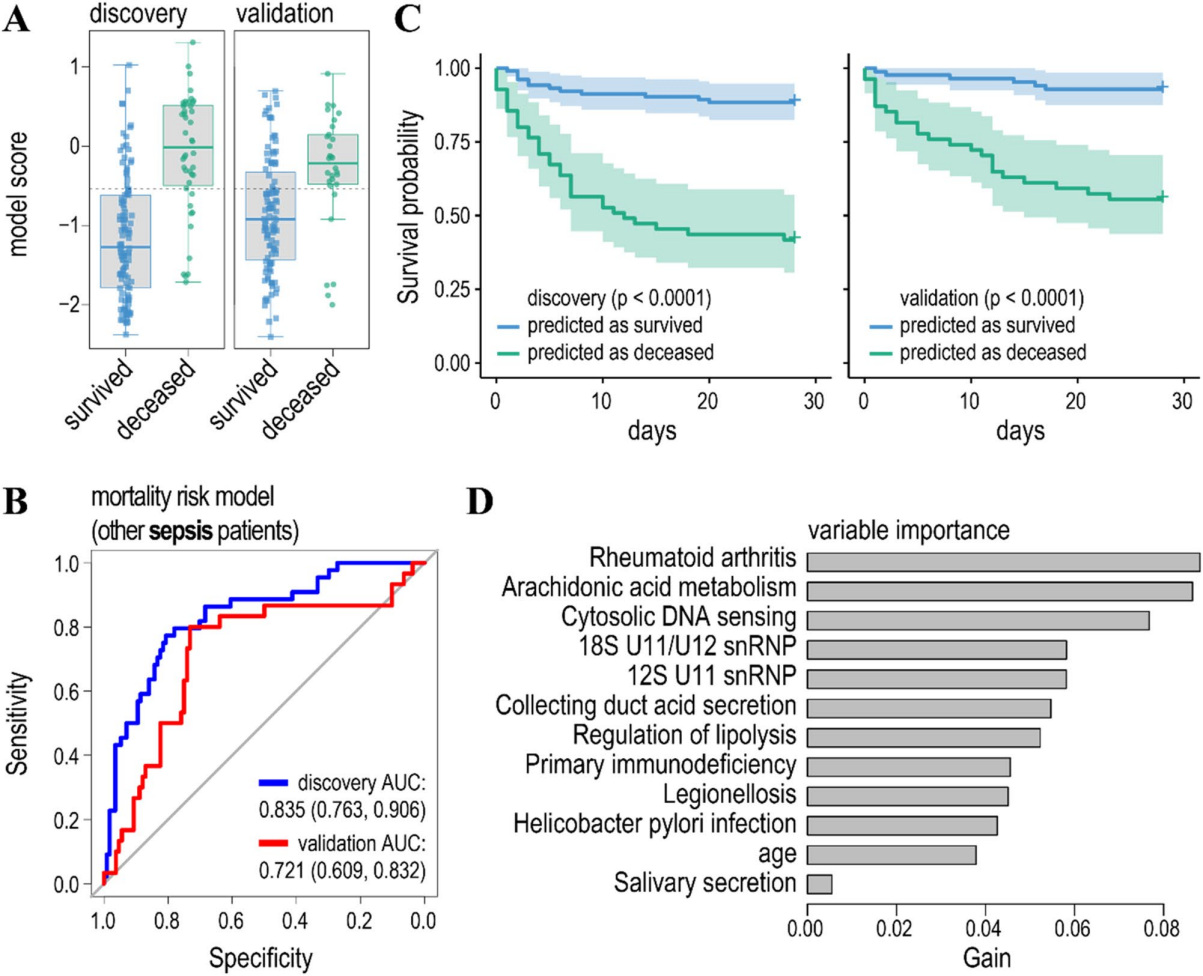
The model predicting mortality/survival of other sepsis patients. (**A**) Boxplots of the model scores. (**B**) ROC curves for the model predicting mortality in sepsis patients. Cohorts were partitioned as in the previous study^8^. (**C**) Survival probability for the patients predicted to have low (blue line) and high (green line) risk of mortality. (**D**) Relative contribution of gene ensemble noise features to the model.

In an attempt to increase the prediction accuracy, we trained separated gradient boosted tree models for sepsis/CAP (Fig. 4) and sepsis (Fig. 5) patients. Indeed, in both cases the accuracy of the prediction of the minor class (deceased patients) increased (Table 2 and S2) in both the discovery and validation cohorts. Likewise, AUCs for the validation cohorts were also higher as compared to the model predicting mortality for both cohorts of sepsis patients (compare Fig. 4B and 5B with Fig. 3B). To that, differences in AUCs between discovery and validation cohorts were lower for the models predicting mortality separately for sepsis/CAP and sepsis patients as for the model trained on both types of patients. This was especially evident for the model predicting mortality for the sepsis/CAP patients (Fig. 4B). Thus, knowing the cause of sepsis improves the prediction accuracy of the models.

Finally, it has to be noted that both the feature selection and gradient boosted regression trees allow for the ranking of the model features’ importance (Figs. 3D, 4D, 5D, and Table S3). First, it turned out that a patients’ age does not noticeably contribute to the prediction of mortality in sepsis/CAP patients and it ranks low in the prediction of mortality of sepsis patients. Second, high ranking gene ensembles (pathways) could immediately be associated with the host response to infection and, thus, pathology of the sepsis. These include legionellosis (a pathway responsible for atypical pneumonia caused by *Legionella* bacteria), epithelial cell signalling in *Helicobacter pylori* infection and leishmaniasis, and imbalances in these pathways either caused by corresponding infections or immune activation could lead to sepsis^57–59^. To that, gene ensemble noise in immune pathways, such as rheumatoid arthritis and primary immunodeficiency, contribute to the prediction of clinical outcome in sepsis patients (Fig. 3D, 5D, and Table S3).

### Weighted correlation network analysis of sepsis patients

As an alternative to the gene ensemble noise, we considered weighted correlation network analysis (WGCNA) to predict clinical outcomes for the sepsis patients. WGCNA clusters genes into distinct modules based on the co-expression adjacency and topological overlap, and projects modules to the first principal component—eigengene^60^. Identification of the co-expression modules for the CAP and sepsis patients revealed only a weak linear association of WGCNA eigengenes with the risk of mortality. The eigengene values were compared between survived and deceased CAP and sepsis patients with t-tests followed by FDR adjustment of *p*-values (Figure S5). Next, we trained the XGBoost classification models to predict the mortality of sepsis patients using WGCNA eigengenes as explanatory variables. Interestingly, the resulting classification accuracies of these models were comparable to the models based on the gene ensemble noise features (Table 2, Figure S6, Table S2). This suggests that WGCNA eigengenes contribute to the risk of mortality non-linearly as XGBoost decision tree ensembles are capable of incorporating non-monotonic associations in the model. As with any DGE-type of analysis, WGCNA modules require GSEA for biological interpretation. We annotated the modules ranking above the set of unassigned genes (module 0) by the variable importance for each model using KEGG and CORUM gene annotations. A standard hypergeometric test revealed a number of significant enrichments for KEGG/CORUM annotations, including mitochondrial respiratory chain complex I (Complex I) for some of the WGCNA modules including (Figures S7, S8, S9). However, a bulk of WGCNA modules lacked any significant KEGG/CORUM enrichments pointing to the limitations of biological interpretability of WGCNA. Gene ensemble noise voids this limitation as it operates directly on the level of known biological ensembles (pathways, protein complex subunits, etc.) instead of individual genes or eigengenes.

### Gene ensemble noise and WGCNA predict the severity of the COVID-19 disease and uncover pathways for pharmaceutical targeting

Considering the impact of the COVID-19 pandemic and the availability of leukocyte RNA sequencing data for the COVID-19 patients^61^, we replicated the gene ensemble noise and WGCNA analysis to grasp more insights into the immune response to the SARS-CoV-2 virus. The 100 COVID-19 patients were assigned into two equally-sized groups: severe and less-severe (mild), based on the ‘‘hospital-free days at day 45’’ (HFD-45). The severe group corresponds to the patients with HFD-45 < 26 and the less-severe to HFD-45 ≥ 26. For each group, we assessed the biological coefficients of variations in genes’ RNA counts ($bcv^{2}$) using the negative-binomial (NB) model. To this end, we applied GAMLSS (generalized additive model for location, scale and shape) to evaluate the statistical impact of the severity of the COVID-19 disease (fixed effect) on both parameters of the NB distribution ($\mu$—mean expression and $bcv^{2}$). Age and gender were incorporated into the model of $\mu$ as random effects. For the details of GAMLSS and its application to the statistical inference of genes’ $bcv^{2}$ see^20,29^. Similar to H1N1 and sepsis/CAP, the inter-individual variability of leukocyte gene expression increased in the severe COVID-19 patients (compare Fig. 1A with 6A). This indicates that both viral and bacterial infections cause a heterogenous immune response manifested in the increased $bcv^{2}$ of RNA copy numbers.

**Figure 6:**
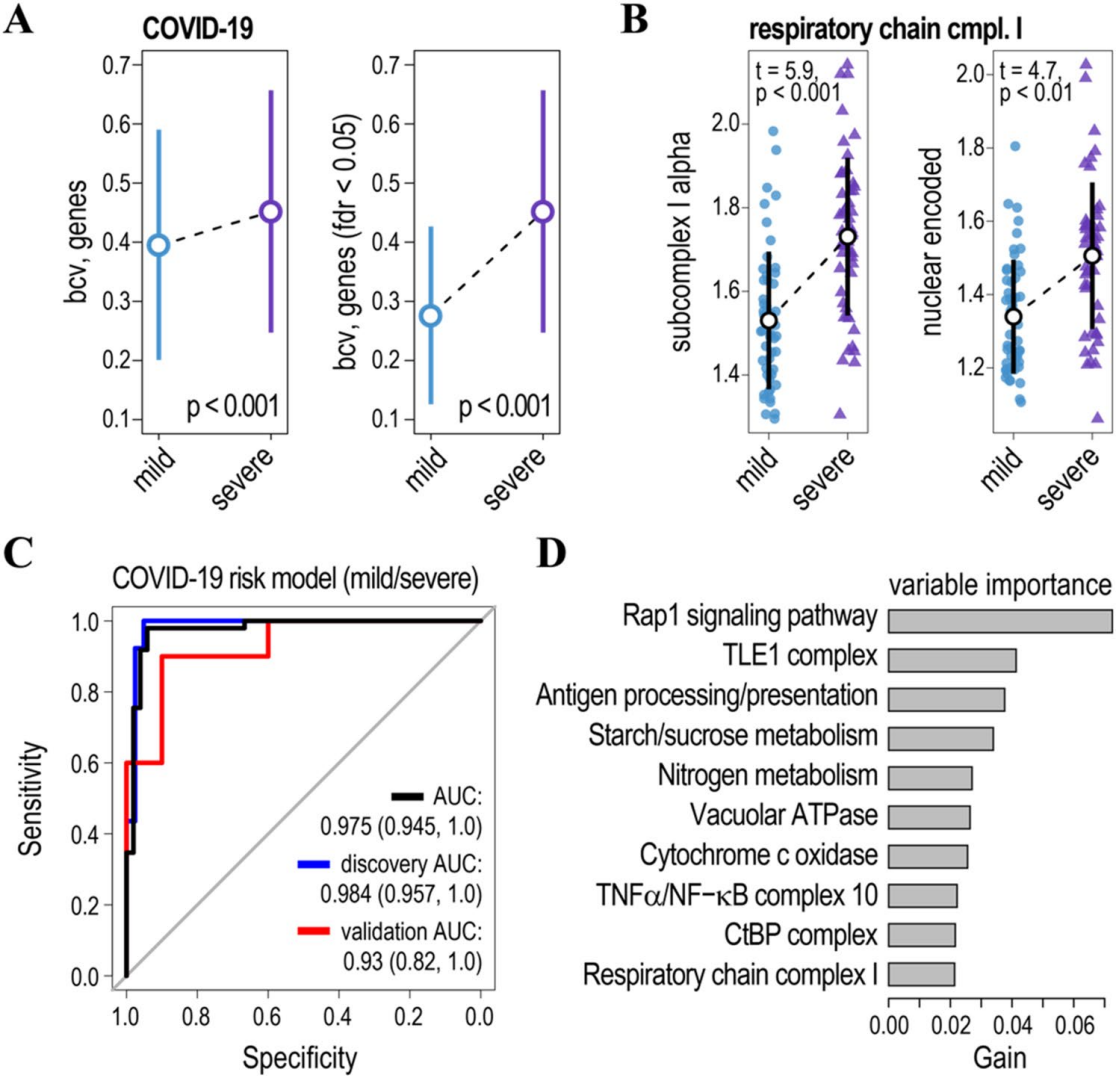
Association of gene ensemble noise with the COVID-19 disease state. (**A**) Inter-individual biological variability in leukocyte gene expression (bcv—biological coefficient of variation) increases in severe COVID-19 patients as compared to the mild ones. Left panel—average estimates of the bcv for all genes expressed in the patients’ leukocytes, right panel—bcv for the genes with significant changes in inter-individual biological variability (false discovery rate, FDR < 0.05). *p*-values of t-tests comparing differences in inter-individual gene expression variability for mild and severe COVID-19 patients. Circles and whiskers indicate means and standard deviations respectively. (**B**) Plots of gene ensemble noise for genes encoding subunits of mitochondrial respiratory chain complex I (subcomplex I alpha—left panel and nuclear-encoded subunits—right panel). Black circles and whiskers indicate means and standard deviations. t and *p*-values of the tests comparing gene ensemble noise for mild and severe COVID-19 patients are shown. (**C**) ROC curves for the model based on the gene ensemble noise for the discovery (blue line) and validation (red line) cohorts, and all samples (black line). For further details on the model accuracy see Tables 2 and S4. (**D**) Relative contribution of gene ensemble noise features to the model. For further details see Table S5.

Analysis of the gene ensemble noise revealed a significant impact of the COVID-19 disease on many KEGG and CORUM annotated ensembles (Figs. 2A, 6B, Figure S10). Moreover, COVID-19, H1N1, and sepsis exert a common impact on gene ensemble noise of respiratory chain complex I, HIF-1 signalling, peroxisome, necroptosis, NOD-like receptor, Fc epsilon RI signalling pathways, and so on (Fig. 2A) suggesting similar perturbations in stoichiometry/gene noise of these gene ensembles caused by an immune response. This, in turn, indicates that pharmaceutical targeting of these ensembles could lead to the development of common first-line treatments in response to present and future pandemics. Interestingly, respiratory chain complex I was also identified as being associated with the COVID-19 disease state (mild/severe) in the analysis of WGCNA modules (Figure S11). Thus, both approaches taking completely different analysis routes converged on the same target (Complex I) substantiating its role in the COVID-19 disease.

Finally, we trained the gradient boosted regression tree models to classify the severity of the COVID-19 disease using either gene ensemble noise or WGCNA eigengenes as explanatory variables. To this end, we randomly split the data into 80% (80 patients) discovery and 20% (20 patients) validation cohorts to replicate the multi-omics COVID-19 study design^61^ and trained the XGBoost models. The model based on the gene ensemble noise accurately classified mild and severe COVID-19 patients in both discovery and validation cohorts (Fig. 6C, Figure S12A, Table 2 and Table S4). As such, the overall model’s accuracy (Sensitivity, Specificity, AUC, etc.) was comparable to the models based on the multi-omics transcriptome, proteome, and metabolome profiles^61^. Ranking of the gene ensemble noise variable importance for the model (Fig. 6D, Table S5) revealed several regulators of inflammation: TLE1 corepressor complex, CtBP complex and TNFα/NF-κB complex 10^62,63^. In addition, variable importance of cytochrome c oxidase and nitrogen metabolism gene ensembles also ranked high (Fig. 6D, Table S5). Nitrogen metabolism is dysregulated in the COVID-19 patients^64^, while alterations in cytochrome C ensemble noise along with the respiratory chain complex I further point to mitochondrial respiration disfunction.

The accuracy of the model trained with the WGCNA set of explanatory variables was comparable to the model based on gene ensemble noise (Figure S12A,B, Table 2, and Table S4). Ranking of the WGCNA eigengenes variable importance followed by the KEGG/CORUM enrichment analysis further confirmed an association of the respiratory chain complex I with the severity of the COVID-19 disease (Figure S12C,D).

Altogether, we conclude that the gene ensemble noise provides novel approaches and insights to the discovery of biomarkers, prediction of clinical outcome, and the molecular mechanisms of pathology from the point of view of imbalances in stoichiometry and gene noise in the expression of biologically interpretable gene ensembles.

## Discussion

Here we attempted a dissection of molecular mechanisms of human pathology, exemplified by SARS-CoV-2 and H1N1 infection, and sepsis, through a prism of gene ensemble noise. Unlike classical DGE, gene ensemble noise allows for the identification of imbalances in the expression of entire gene circuits, rather than individual genes on the level of stoichiometry and gene noise. This approach offers an alternative, but non-mutually exclusive to the DGE interpretation of a molecular basis of disease and both have their own strengths and weaknesses. At the same time identification of the same targets by both approaches only strengthens confidence in the findings, as exemplified here by comparison of the results of the gene ensemble noise and WGCNA analysis.

We noted in the introduction that due to a fluctuation-response a statistical inference of DGE might be biased towards genes with high inter-individual variability, *i.e.* “noisy” genes^18,19^ (Figure S1). However, the same applies to gene ensemble noise (Figure S4). This imposes a certain problem to the interpretation of both DGE and gene ensemble noise. On one hand, it can be suggested that large deviations in the expression of genes and ensembles, which are naturally prone to high fluctuations, might not be causative for a disease, as an organism is already adapted to such variations. On the other hand, these genes/ensembles themselves might play an important adaptive role^65^ and their over-response could lead to disease. At the moment it seems difficult to come to a resolution between these two possibilities, but they should be considered, specifically in the identification of pharmaceutical targets: genes or gene ensembles (pathways, protein complexes).

As compared to DGE, gene ensemble noise provides a holistic interpretation of mis-regulation in gene expression under pathologic or other conditions. As it operates on the level of gene ensembles it does not require gene set enrichment analysis (GSEA), thus it circumvents potential pitfalls of GSEA associated with the cut-off problem of DGE^24,25^. As with any gene expression analysis, gene ensemble noise relies on the quality and completeness of pathways and the protein complexes’ annotation. Finally, we noted that inter-individual variability of gene ensemble noise is significantly less than that of individual gene expression (Figure S3). This, in turn, might improve the accuracy of diagnostic and clinical outcome models. Though it might come at the expense of few features being available for the selection and training of models. At the same time, in future studies, both DGE and gene ensemble noise could be combined.

In this study, we applied the concept of gene ensemble noise to the analysis of critically ill H1N1, and sepsis patients^8,28^, as well as to the COVID-19 disease^61^. We noted a large-scale gene response in two dimensions: on the level of mean gene expression and the level of variance (inter-individual variability). Interestingly, both responses were correlated (Fig. 1D) and both were dependent on gene variance suggesting that the fluctuation-response might drive changes in these two parameters of gene expression co-ordinately^18^. In all cases (COVID-19, H1N1, and sepsis), inter-individual variability was increased for a bulk of the genes. Consequently, we only identified pathways or gene complexes for which gene ensemble noise was significantly increased for H1N1, and sepsis patients as compared to healthy individuals, and for the COVID-19 as compared between severe and less-severe patients. It is interesting to note that dysregulation of certain pathways is commonly seen and is informative for prediction of patient survival across diseases. Thus, uncoordinated response to atypical pneumonia caused by *Legionella* bacteria as well as epithelial cell signalling in *Helicobacter pylori* infection and leishmaniasis might also highlight general deficiency in immune response resulting in higher vulnerability of sepsis patients. Our results suggest that inter-individual gene expression variability is a prominent driver of gene ensemble noise in these patients.

Because viral/bacterial infections and sepsis result in an overwhelming gene expression response, it is difficult to identify a reasonably small set of genes or gene ensembles for biological interpretation. Thus, we only focused on the pathways (protein complexes) for which gene ensemble noise increased in all cases and correlated these with a disease state (Fig. 2A). From this intersection, we inferred 13 pathways, most of which have been previously implicated in sepsis (Table 1). To that, 5 pathways (protein complexes) showed a significant association of gene ensemble noise with the severity of COVID-19, H1N1 infection phase, and sepsis (CAP, other) mortality risk (Fig. 2A). Potentially, these pathways could be targeted for adjuvant treatment of sepsis. Especially, we consider mitochondrial respiratory chain complex I (Complex I) (Fig. 2D) and peroxisome promising for pharmaceutical targeting.

Increased gene ensemble noise for the Complex I would imply either altered stoichiometry or increased gene expression noise for genes encoding subunits of the Complex I or both. As a result, this might lead to an improper assembly of Complex I resulting in dysregulation of mitochondrial respiration and the manifestation of the Warburg effect. Interestingly, the Warburg effect is one of the hallmarks of sepsis and other inflammatory diseases^66^. Likewise, the mitochondrial disfunction was also evidenced from the recent multi-omics analysis of the COVID-19 disease as a significant decrease in the citrate levels was noticed in the severe COVID-19 patients^61^. The impaired Complex I function can be bypassed by an alternative redox mediator, such as methylene blue^50,51^. To that, methylene blue is a selective inhibitor of the nitric oxide–cyclic guanosine monophosphate (NO–cGMP) pathway^53^, and increased NO levels are a hallmark of sepsis^67^. Some clinical studies have already indicated a beneficial role of methylene blue in the treatment of sepsis^52,53^. Similar to mitochondrial respiration, peroxisomes also play an important role in the pathology of sepsis as the dysfunction of peroxisomes results in oxidative stress^32^. Again, an increased gene ensemble noise for the peroxisome pathway indicates a potential mechanism for such dysfunction in H1N1, and sepsis patients. Potentially peroxisome biogenesis could be restored by 4-phenylbutyrate and there several studies indicating its positive role in the treatment of sepsis^68,69^. Considering future directions, it could be proposed that the search for epigenetic modulators of gene ensemble noise might represent a novel pharmaceutical avenue for adjuvant treatments of sepsis.

Finally, we explored the possibility to use gene ensemble noise in the prediction of clinical outcomes. Previously some promising biomarkers and gene expression endotypes associated with septic shock and mortality have been identified based on DGE analysis^8,15^. However, as already mentioned, gene ensemble noise looks at gene expression from a different, yet complementary, angle, thus enabling the identification of novel pathways and biomarkers for sepsis and other diseases. To that, models predicting pathology based on gene ensemble noise could potentially be more robust, as inter-individual variability for gene ensemble noise is lower than that for log gene expression (Figure S3). Furthermore, gradient boosted regression tree models trained on sepsis patients to predict their mortality had a good accuracy on the validation cohort (Fig. 3, Table 2). These outperformed predictions based on the Mars1 gene expression endotype, which was shown to associate with a poor prognosis^8^, both on the discovery and validation cohorts (Fig. 3C). Interestingly, some gene ensemble noise features selected statistically for the models predicting mortality in both cohorts of sepsis patients could immediately be related to the host’s response to infection. For example, increases in gene ensemble noise in legionellosis, epithelial cell signalling in *Helicobacter pylori* infection, and leishmaniasis pathways could potentially serve as biomarkers of sepsis and its outcome.

We also attempted to improve models’ accuracies by training multi-layer perceptrons (MLP), a vanilla deep-learning approach, as well as convolutional neural networks (CNN) with Keras (https://keras.io). However, the balanced accuracies of the MLP and CNN models for the validation cohorts were lower than that of the gradient boosted regression trees (data not shown), which might be due to a small-size learning sample problem^70^. Nonetheless, deep-learning could represent a powerful approach for the multi-omics analysis in the future as recently CNN models for classification of tumour types based on RNA-seq has been developed^71^.

In conclusion, here we showed the potential of gene ensemble noise in the biological interpretation of a disease, the identification of pharmaceutically targetable pathways, novel biomarkers, and the prediction of clinical outcome. It is worth noting that while we showed applications of gene ensemble noise for sepsis and COVID-19 using biochemical complexes and KEGG pathways, datasets for other diseases and types of ensembles such as gene ontology, common promotor regulatory elements or miRNA sites can also be employed. We believe that gene ensemble noise analysis could be broadly applied alongside DGE to dissect molecular mechanisms of the pathology in two complementary dimensions: in Jacob-Monod dimension of specific gene regulation and a novel dimension of holistic gene circuit regulation.

## Methods

### Data resources and processing

The computer code and the models used in this project are deposited to the following public repository: https://github.com/Vityay/GeneEnsembleNoise.

GSE65682 Affymetrix Human Genome U219 Array whole blood gene expression profiles were used for the analysis of community and hospital-acquired pneumonia (HAP/CAP) sepsis patients (here referred to as sepsis/CAP) and no-CAP sepsis patients (here referred to as other sepsis)^8,12^. In brief, the cohort consisted of 42 healthy individuals (24 males, 18 females), 183 sepsis/CAP patients (111 males, 72 females), and 296 other sepsis patients (161 males, 135 females). The mean age of sepsis/CAP (61.5 ± 1.2) and other sepsis (60.6 ± 0.8) patients did not differ significantly (*t*(350.37) = 0.59, *p* = 0.56), however healthy individuals were significantly younger as compared to sepsis/CAP (*t*(54.0) = 4.7, *p* < 0.001) and other sepsis (*t*(47.2) = 4.6, *p* < 0.001) patients. Out of 183 sepsis/CAP patients, 40 died within 28 days and out of 296 other sepsis patients, 74 died within 28 days. Thus, we divided sepsis/CAP and other sepsis patients into survived and deceased groups, considering these two states as an ordered factor (ordinal) variable (survived < deceased). Overall sepsis/CAP and other sepsis patients were similar in treatment and outcome and other sepsis patients were suspected for CAP and treated with antibiotic (see Scicluna et al.^12^ for the detailed description of the cohorts). For the analysis we used 521 samples out of 802 for the following reasons: (1) to compare our risk of mortality models with the previously published^12^ on the same discovery and validation cohorts and (2) for the remaining 281 there were no survivorship data available.

GSE21802 Illumina human-6 v2.0 expression bead-chip whole blood gene expression profiles were used for the analysis of H1N1 infected patients^28^. The cohort consisted of 4 healthy individuals and 19 H1N1 patients (8 in the early and 11 in the late phase of the disease). The early phase was defined as early, from the onset of symptoms—day 0 to day 8, and late—from day 9 and above. The statistics of the cohorts are given in Table 1 of the original study^28^, however, neither sex nor age assignments were available for the patients from the GSE21802 series annotation. We used 521 out of 802 for the following reasons: (1) to compare our risk of mortality models with the previously published study by Scicluna, et al. 2015 on the same discovery and validation cohorts and (2) for the remaining 281 there were no survivorship data available. GSE157103 leukocyte RNA-seq gene expression profiles were used for the analysis of the COVID-19 disease. The cohort consisted of 100 COVID-19 patients split into less-severe (mild) and severe groups based on the hospital-free days as described^61^.

GSE65682 microarrays signal intensities were pre-processed (background corrected and RMA-normalized) with the Bioconductor *oligo* package^72^. Lowly-expressed and outlier genes were identified in high dimensions using the spatial signs (*sign2*) algorithm of *mvouliter* R package with a critical value for outlier detection at 0.9. The robust principal components explained variance of 0.95^73^. GSE21802 signal intensities significantly above the background were quantile normalized^74^. Genes were annotated with Bioconductor *hgu219.db* and *illuminaHumanv2.db* database packages for GSE65682 (8826 genes) and GSE21802 (7240 genes) respectively. GSE157103 RNA-seq counts were analysed with GAMLSS as previously described^20^.

### Statistical analysis of gene expression variability and gene ensemble noise

Statistical analysis was done using R and R/Bioconductor packages.

To estimate the inter-individual gene expression variability for healthy and sepsis patients we accounted for age as a random effect. To this end, we used Generalized Additive Model for Location, Scale, and Shape (GAMLSS)^20,29^. In brief, for normally distributed log-transformed microarray intensities ($Y = log\left( X \right)$, $Y \sim N\left( {\mu_{Y} ,\sigma_{Y} } \right)$), GAMLSS allows for the modelling of both parameters of gene expression (mean and dispersion):

$$\mu_{Y} \sim D\beta_{\mu } + Zu_{\mu } ,$$

$$log\left( {\sigma_{Y} } \right) \sim D\beta_{\sigma } + Zu_{\sigma } ,$$

where $\mu_{Y} = \left( {\mu_{{Y_{1} }} , \ldots ,\mu_{{Y_{n} }} } \right)^{T}$ and $\sigma_{Y} = \left( {\sigma_{{Y_{1} }} , \ldots ,\sigma_{{Y_{n} }} } \right)^{T}$ are the vectors of means and dispersions for $Y = \left( {Y_{1} , \ldots ,Y_{n} } \right)$. $D$ − $n \times p$ fixed effect design matrix for the disease state (healthy, survived, deceased). $\beta_{\mu } = \left( {\beta_{{\mu_{1} }} , \ldots ,\beta_{{\mu_{r} }} } \right)^{T}$ and $\beta_{\sigma } = \left( {\beta_{{\sigma_{1} }} , \ldots ,\beta_{{\sigma_{r} }} } \right)^{T}$—estimated fixed effect coefficients for mean and dispersion. $Z$ − $n \times k$ random effect design matrix for age (age was binned into 10 deciles). $u_{\mu } = \left( {u_{{\mu_{1} }} , \ldots ,u_{{\mu_{k} }} } \right)^{T}$ and $u_{\sigma } = \left( {u_{{\sigma_{1} }} , \ldots ,u_{{\sigma_{k} }} } \right)^{T}$—estimated random effect coefficients for mean and dispersion, where $u \sim N\left( {0,\delta } \right)$. With GAMLSS it is also straightforward to test for the significance of a factor effect on either the mean, the variance, or both with likelihood ratio test^20^.

Gene ensemble lists were generated by the mapping of genes to the KEGG-annotated biological pathways or CORUM-annotated subunits of mammalian protein complexes^22,23^. Their gene noise was estimated for each individual by calculating the variances of log-transformed expressions of genes for each ensemble (Fig. 1S). Estimates of gene ensemble noise were correlated with the disease states (healthy < early phase < late phase for H1N1 and healthy < survived < deceased for sepsis) by Kendall rank correlation, treating the disease state as an ordinal variable. Linear trends between disease states and gene ensemble noise were estimated by rank-based regression^75^.

### Gradient boosted regression tree models

To predict the mortality of sepsis patients we trained gradient boosted regression tree models with a scalable tree boosting system XGBoost^54^ using mortality within 28 days as a binary response variable, and gene ensemble noise and age as independent model features. To this end, we split individuals into discovery and validation cohorts following the same partitioning as annotated in GSE65682. Then, we trained 3 models: (1) a model predicting mortality for all sepsis patients, (2) a model predicting mortality for sepsis/CAP patients, and (3) a model predicting mortality for other sepsis patients. Model features were preselected using discovery cohorts by *t*-test comparing gene ensemble noise for survived and deceased patients to maximize the accuracy of XGBoost training on the discovery data sets. For all-cause sepsis (1), and non-CAP sepsis (3) models, the cut-off for the model features was set at *p* ≤ 0.01, and for the sepsis/CAP model (2)—at *p* ≤ 0.05. The XGBoost hyper tuning parameters (learning rate (η), complexity (γ), depth, etc*.*) were optimized by cross-validation. To avoid overfitting, we found early epoch stopping parameters by randomly splitting the discovery cohort into two equal folds: training and test. Then, the validation cohorts, which were hidden from feature selection and model training, were used to verify the accuracy of the final models.

## Supplementary information

Supplementary Information.
