# Supplementary material for: Estimates of gene ensemble noise highlight critical pathways and predict disease severity in H1N1, COVID-19 and mortality in sepsis patients
Source: Sci Rep. 2021 May 24;11:10793. doi: 10.1038/s41598-021-90192-9 (PMC8144599; doi:10.1038/s41598-021-90192-9)
Supplement: Supplementary file 1 — Supplementary Information. [file 41598_2021_90192_MOESM1_ESM.docx]

**Supplementary materials**

**Estimates of gene ensemble noise highlight critical pathways and predict disease severity in H1N1, COVID-19 and mortality in sepsis patients**

Tristan V. de Jong^1,4^, Victor Guryev^1,4,*^, Yuri M. Moshkin^2,3,4,*^

^1^ European Research Institute for the Biology of Ageing, University of Groningen, University Medical Centre Groningen, Groningen, The Netherlands,

^2^ Federal Research Centre, Institute of Cytology and Genetics, SB RAS, Novosibirsk, Russia,

^3^ Institute of Molecular and Cellular Biology, SB RAS, Novosibirsk, Russia,

^4^ Gene Learning Association, Geneva, Switzerland

* To whom correspondence should be addressed: E-mail: [yury.moshkin@gmail.com](mailto:yury.moshkin@gmail.com); Tel: +31 6 5272 4873; Fax: +31 50 361 7300; E-mail: [v.guryev@umcg.nl](mailto:v.guryev@umcg.nl)


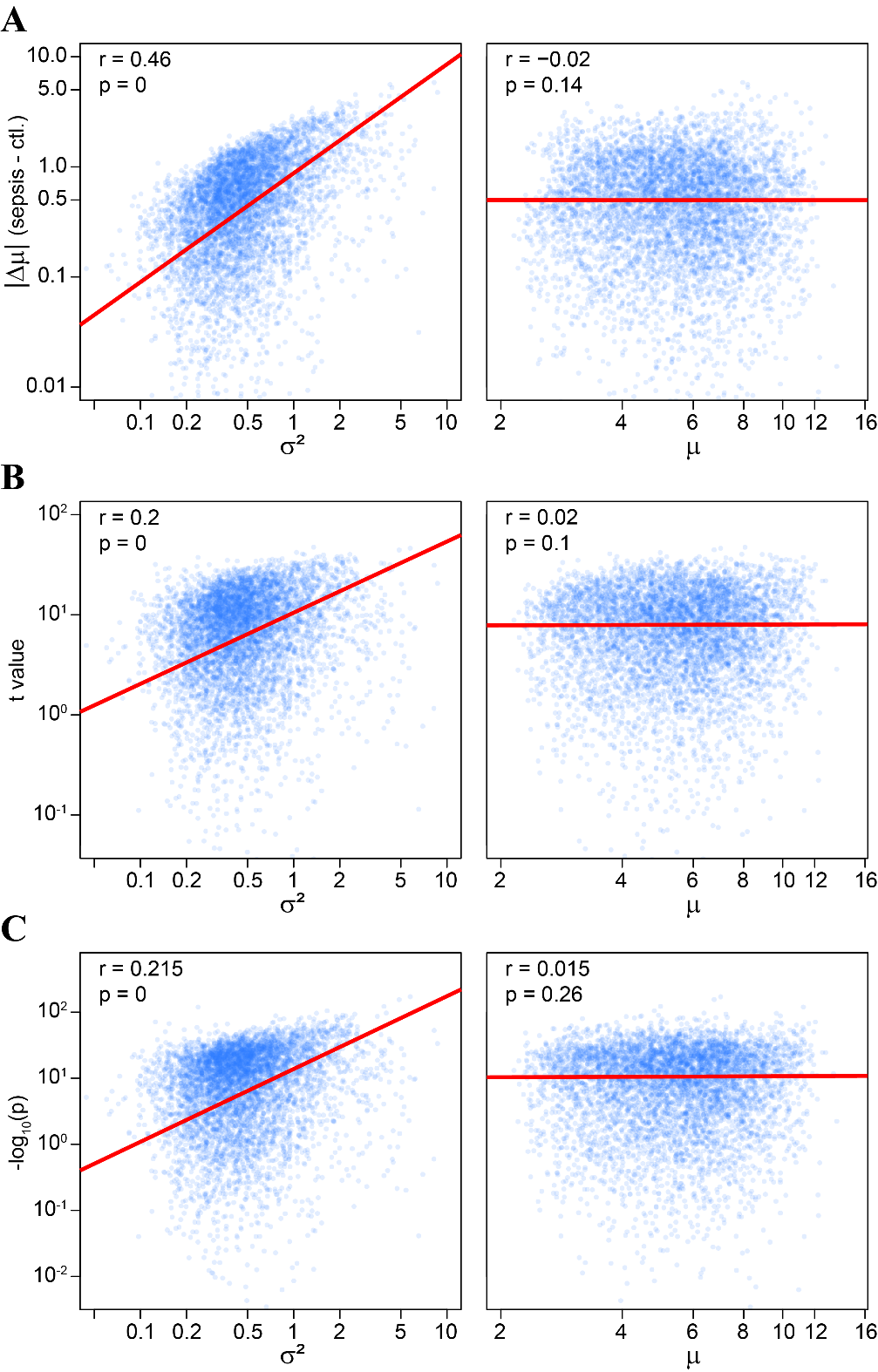


**Figure S1.** **Fluctuation-response relation biases statistical inference of DGE.** **A)** The difference of means of log-transformed bell-shaped gene expression values ($Y=log\left( X \right)$) are proportional to the variance or the squared coefficient of variation (*cv^2^*) of the untransformed variable ($X$): $\left| \bar{Y}_{1}-\bar{Y}_{0} \right|\sim\sigma_{Y}^{2}\approx\frac{\sigma_{X}^{2}}{\bar{X}^{2}}={cv}_{X}^{2}\approx{bcv}^{2}$. Considering RNA copy number ($X$) to be mixed-Poisson (negative-binomial as a specific case) random variable, for large $X$, ${cv}_{X}^{2}$ approximates overdispersion parameter or the biological coefficient of variation (*bcv^2^*) ([de Jong et al. 2019](#_ENREF_14)). A scatterplot on the left panel illustrates fluctuation-response relation (a correlation between absolute differences and variances of log-transformed expression values) for whole-blood gene expression profiles of healthy (ctl.) individuals and sepsis/CAP and other sepsis patients. The data has been taken from ([Scicluna et al. 2017](#_ENREF_49)). This relation is monotonic, but non-linear, suggesting a deviation from the linear coupling between fluctuation (pneumonia/sepsis) and gene expression response. There is no correlation between differences and means of log-transformed expression values (right panel). **B-C)** In the presence of fluctuation-response relation statistical inference will be biased. For example, Student’s *t*-test often used to assess DGE will relate positively to a biological coefficient of variation of gene expression as: $t\sim\frac{\left| \bar{Y}_{1}-\bar{Y}_{0} \right|}{\sigma_{Y}}\sim\frac{\sigma_{Y}^{2}}{\sigma_{Y}}=\sigma_{Y}\approx bcv$. A scatterplot on the left panel shows correlation between Student’s *t* statistic and variance (**B**) and Bonferroni-adjusted p values and variance (**C**). There is no correlation of *t* and *p* values with means of log-transformed expression values (right panel).


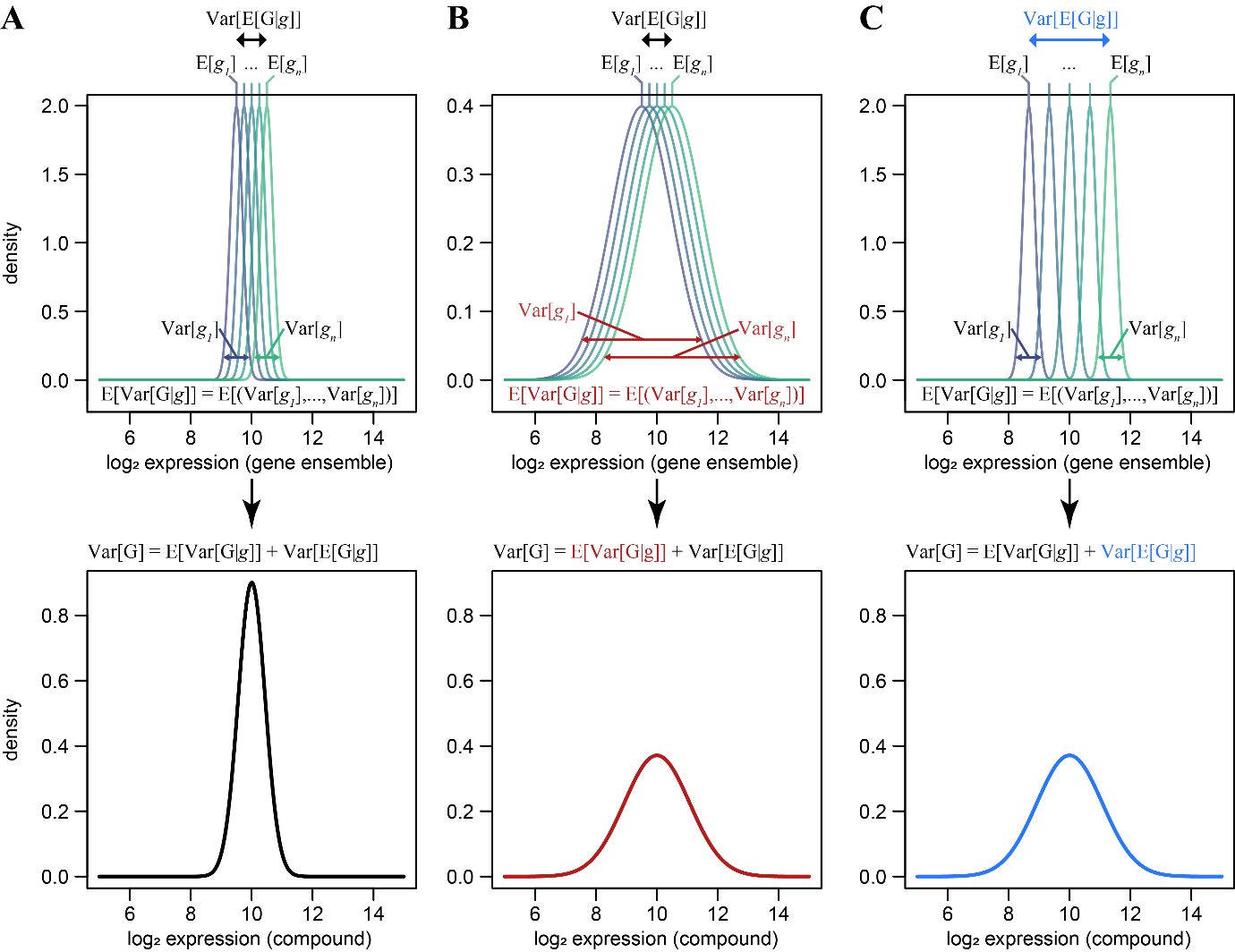


**Figure S2. Gene ensemble noise. A)** Let $G$ be a collection of genes $\left( g_{1}, \ldots, g_{n} \right)$ belonging to either a biological pathway or encoding subunits of a protein complex. Then, from the law of total variance $\mathrm{Var}\left[ G \right]=E\left[ \mathrm{Var}\left[ G|g \right] \right]+\mathrm{Var}\left[ E\left[ G|g \right] \right]$, *i.e.* gene ensemble noise ($\mathrm{Var}\left[ G \right]$) sums from the expected value of genes’ variances ($E\left[ \mathrm{Var}\left[ G|g \right] \right]$) and the variance in genes’ mean expression ($\mathrm{Var}\left[ E\left[ G|g \right] \right]$). The top panel illustrates hypothetical distributions of expressions of genes in an ensemble ($g_{i}$), the bottom panel is derived distribution of gene ensemble ($G=\left( g_{1}, \ldots, g_{n} \right)$). **B, C)** Top panel, changes in variances (**B**) and/or expectations (**C**) of expression of genes will eventually change gene ensemble noise (bottom panel).


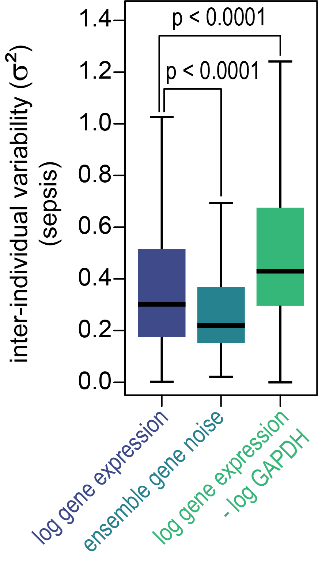


**Figure S3. Comparison of inter-individual variability for log gene expression and gene ensemble noise for sepsis/CAP and other sepsis patients.** Boxplots illustrating population variances for log gene expression, gene ensemble noise, and log gene expression normalized to the GAPDH. Inter-individual variability is significantly less for gene ensemble noise as compared to the log gene expression (according to t-test) and it is higher for GAPDH normalized log gene expressions. The latter follows from the fact that $\mathrm{Var}\left[ log\left( X \right)-log\left( \mathrm{GAPDH} \right) \right]\approx\mathrm{Var}\left[ log\left( X \right) \right]+\mathrm{Var}\left[ log\left( \mathrm{GAPDH} \right) \right]$. Thus, estimating DGE by PCR, which usually requires normalization to some housekeeping gene, results in increased inter-individual variability. Gene ensemble noise can be estimated from PCR without normalization to a reference gene.


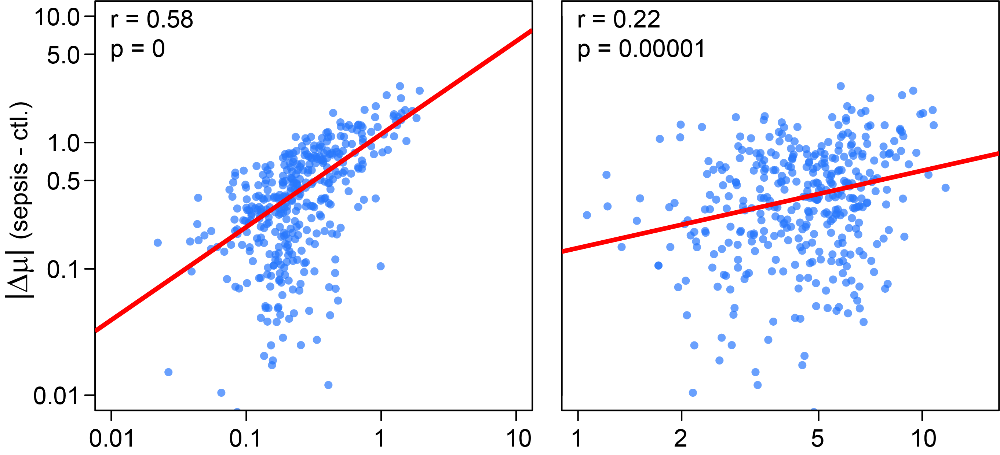


**Figure S4. Fluctuation-response relation for gene ensemble noise.** Differences in means of gene ensemble noise are proportional to the inter-individual variability in gene ensemble noise. A scatterplot on the left panel illustrates fluctuation-response relation (a correlation between absolute differences and variances of gene ensemble noise) for whole-blood gene expression profiles of healthy (ctl.) individuals and sepsis/CAP and other sepsis patients. There is also a modest correlation between absolute changes in gene ensemble noise and the means (right panel).


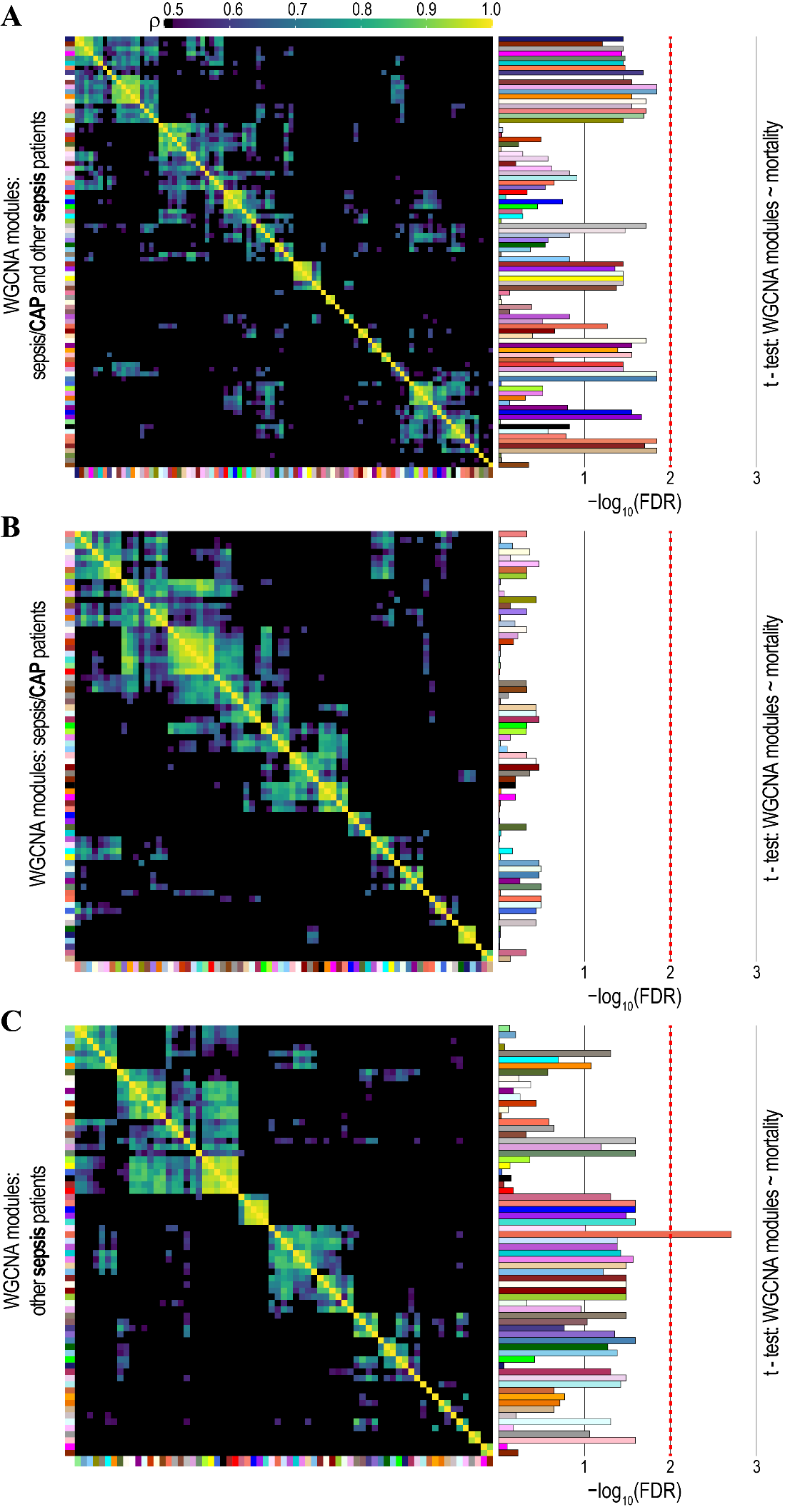


**Figure S5. Weighted correlation network analysis (WGCNA) of associations of eigengenes (modules) with the risk of mortality caused by sepsis.**

**A-C)** Heatmap plots of the correlations in the eigengene networks for the sepsis/CAP and other sepsis patients **(A)**, and sepsis/CAP **(B)** and other sepsis **(C)** patients separately. The color key for the correlations is shown in the top-left. Eigengenes (modules) are labeled by color and are shown on the left side and the bottom of the heatmap. Barplots on the right demonstrate the significance of WGCNA modules (colored bars) associations with the sepsis mortality. -log_10_(FDR) values were derived from the t-tests of WGCNA eigengenes comparing survived and deceased sepsis patients. Note that only one WGCNA module in **(C)** exceeds a stringent significance threshold of -log_10_(FDR < 0.01) > 2 (dashed red lines) indicating an overall only weak monotonic association between WGCNA eigengenes and the risk of sepsis mortality.


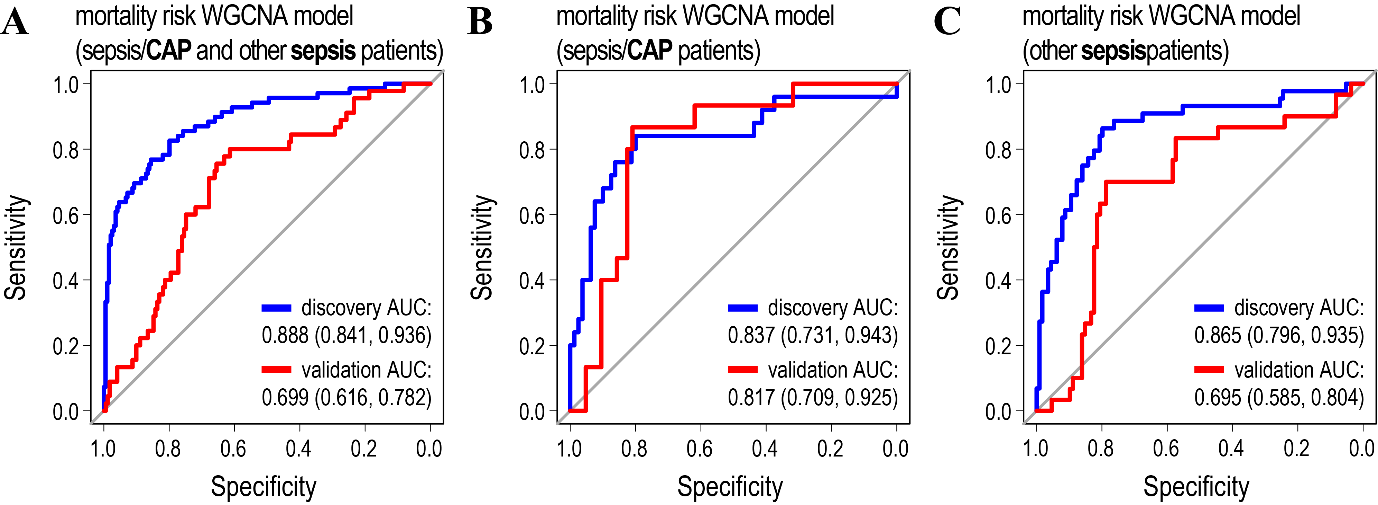


**Figure S6. Models predicting mortality/survival of sepsis/CAP and other sepsis patients based on WGCNA modules.**

**A-C)** Receiver operating characteristic curves (ROC) for the models predicting mortality in sepsis/CAP and other sepsis patients **(A)**, and sepsis/CAP **(B)** and other sepsis **(C)** patients separately. Eigengenes of WGCNA modules were used as explanatory variables. Values for the area under the ROC curve (AUC) are indicated for the discovery (blue line) and validation (red line) cohorts. For further details on WGCNA modules and models’ accuracies see Figures S7-S9 and Tables 2 and S2.


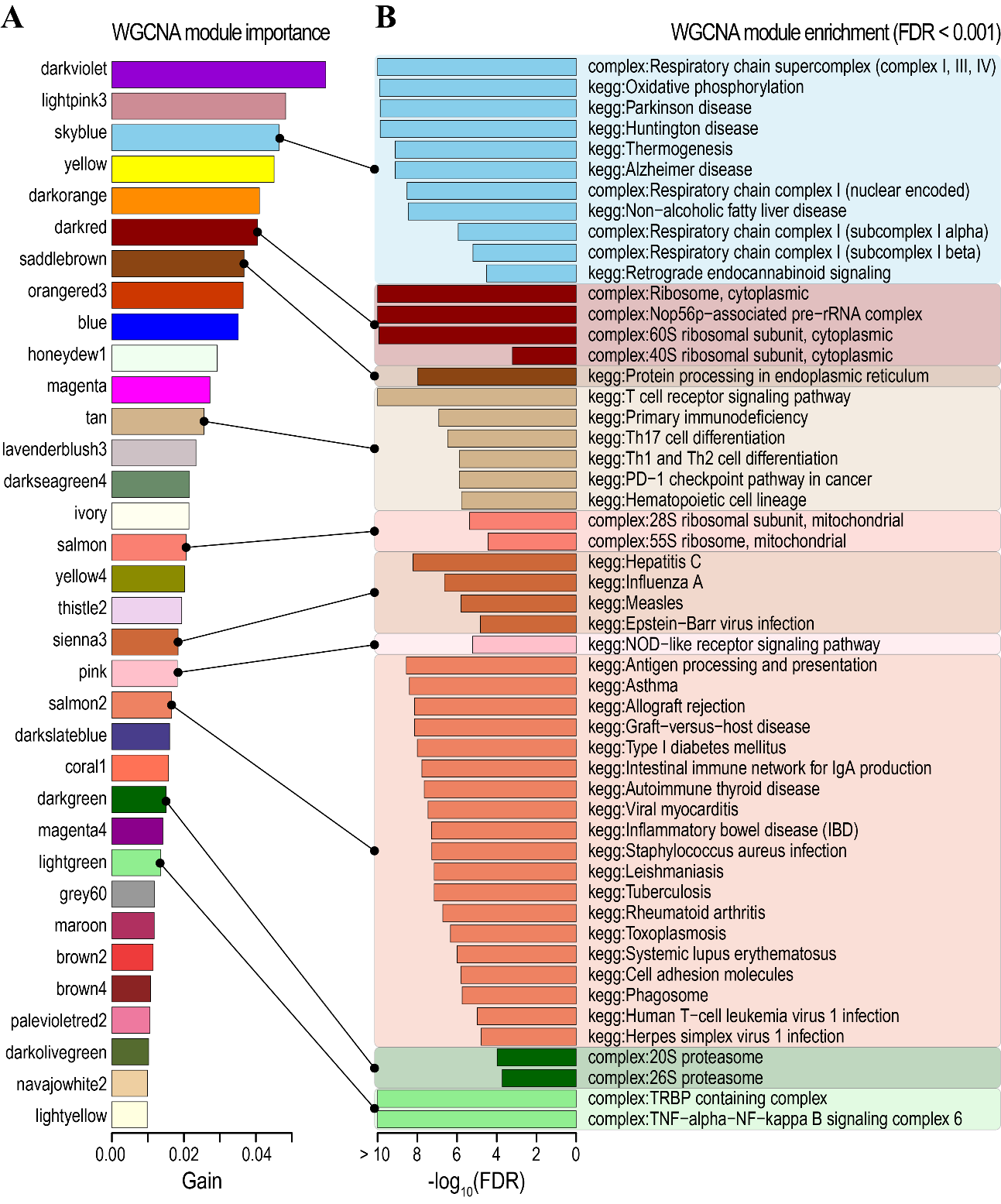


**Figure S7. Annotation of the WGCNA model predicting mortality/survival of the sepsis/CAP and other sepsis patients.**

**A)** Ranking of WGCNA eigengenes (modules) according to their importance (Gain) in the model predicting mortality of the sepsis/CAP and other sepsis patients. Modules with gains exceeding the importance of the set of unassigned genes (module 0) are shown.

**B)** Barplots of the KEGG/CORUM enrichment analysis of WGCNA modules. Each WGCNA module represents a collection of coexpressed/coregulated genes and for each module from **(A)** hypergeometric KEGG/CORUM overrepresentations were estimated. The enriched KEGG and CORUM annotations were selected at FDR adjusted hypergeometric p-value of < 0.001 for each module and shown here on the -log_10_(FDR) scale. Some modules lack any significant KEGG/CORUM enrichments at a given threshold.


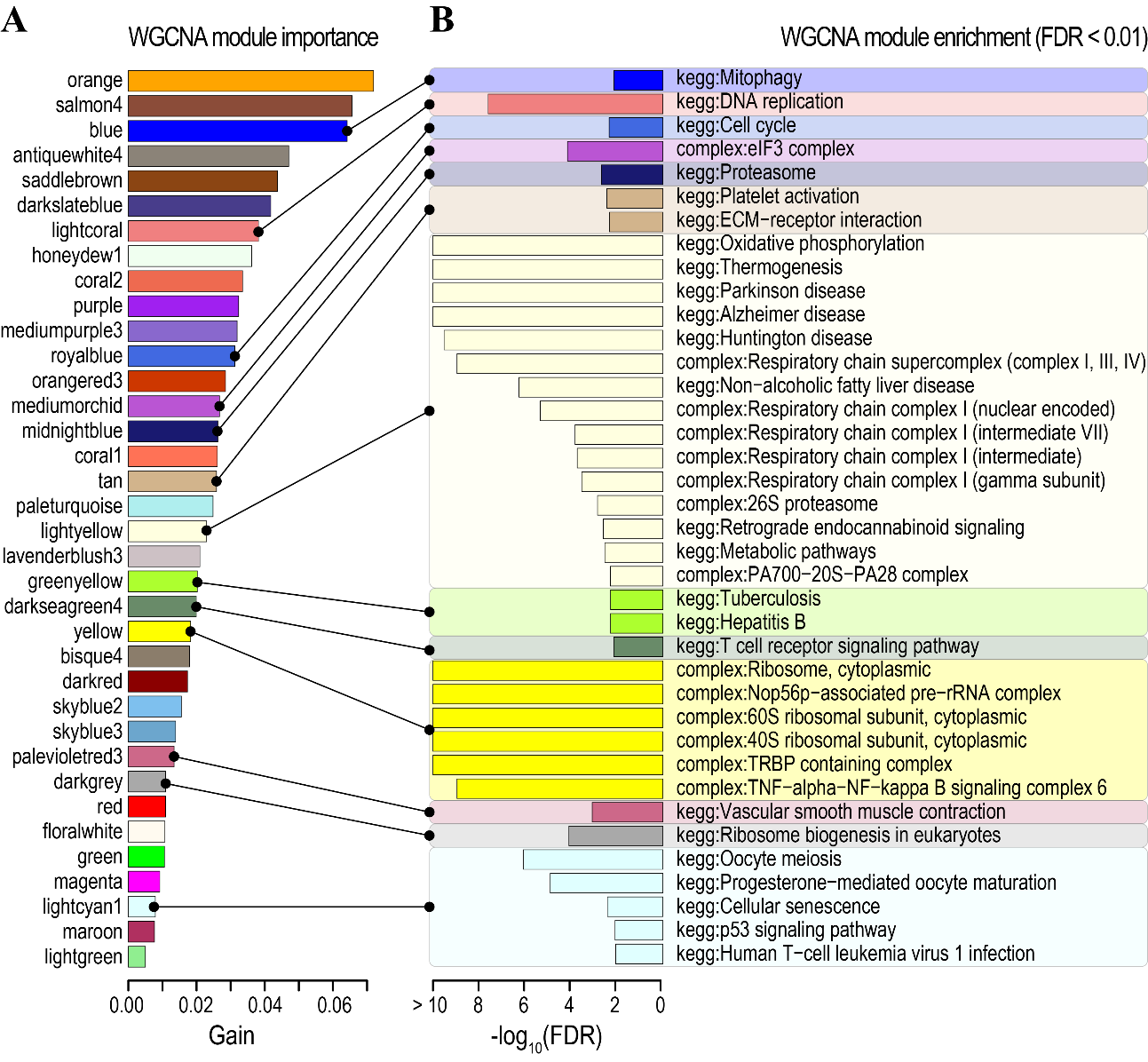


**Figure S8. Annotation of the WGCNA model predicting mortality/survival of the sepsis/CAP patients.**

**A)** Ranking of WGCNA eigengenes (modules) according to their importance (Gain).

**B)** Barplots of the KEGG/CORUM enrichment analysis of WGCNA modules. The enriched KEGG and CORUM annotations were selected at hypergeometric FDR < 0.01.


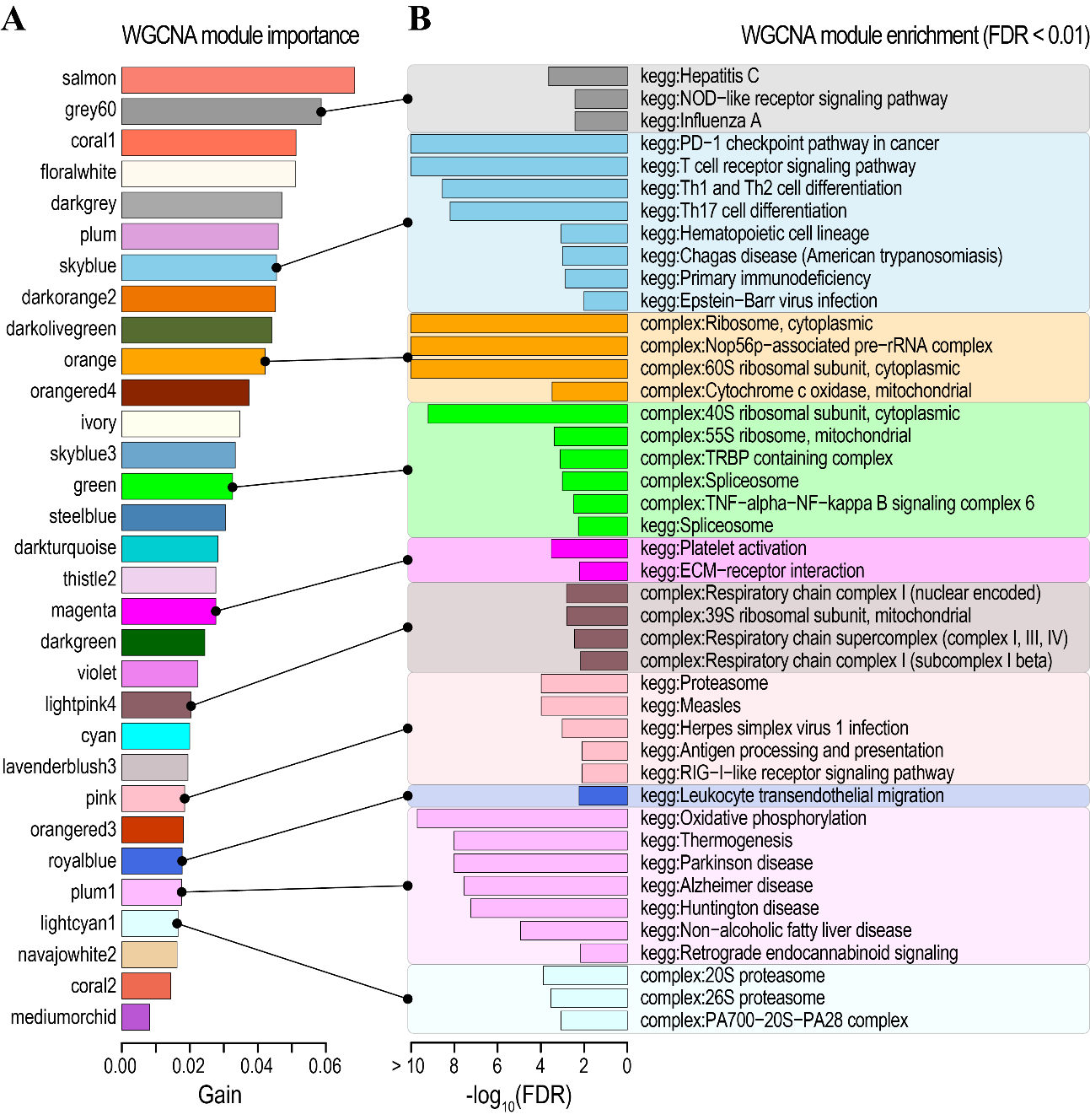


**Figure S9. Annotation of the WGCNA model predicting mortality/survival of the other sepsis patients.**

**A)** Ranking of WGCNA eigengenes (modules) according to their importance (Gain).

**B)** KEGG/CORUM annotation of WGCNA modules. The enriched KEGG and CORUM were selected at hypergeometric FDR < 0.01.


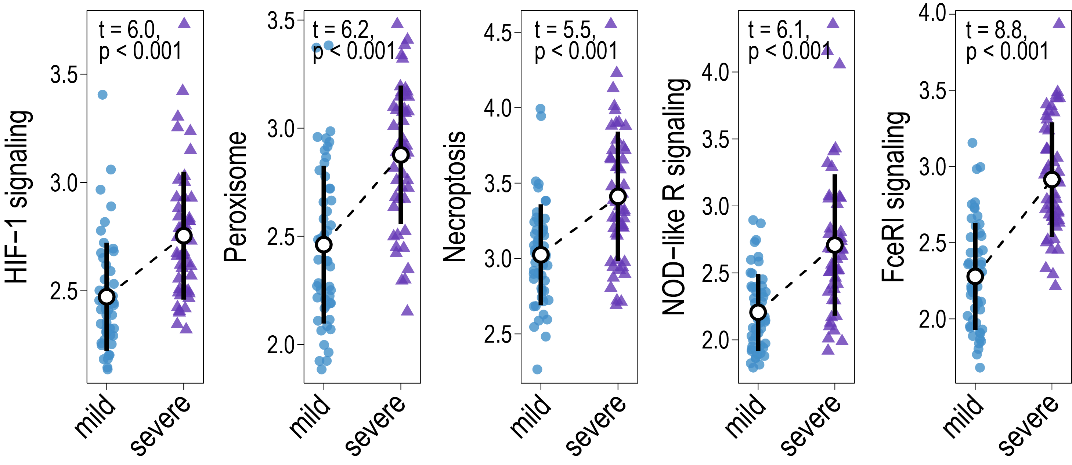


**Figure S10. Association of gene ensemble noise with the COVID-19 disease state for selected pathways.** Plots of gene ensemble noise for genes involved in HIF-1 signalling, peroxisome, necroptosis, NOD-like receptor, and Fc epsilon RI signalling pathways.


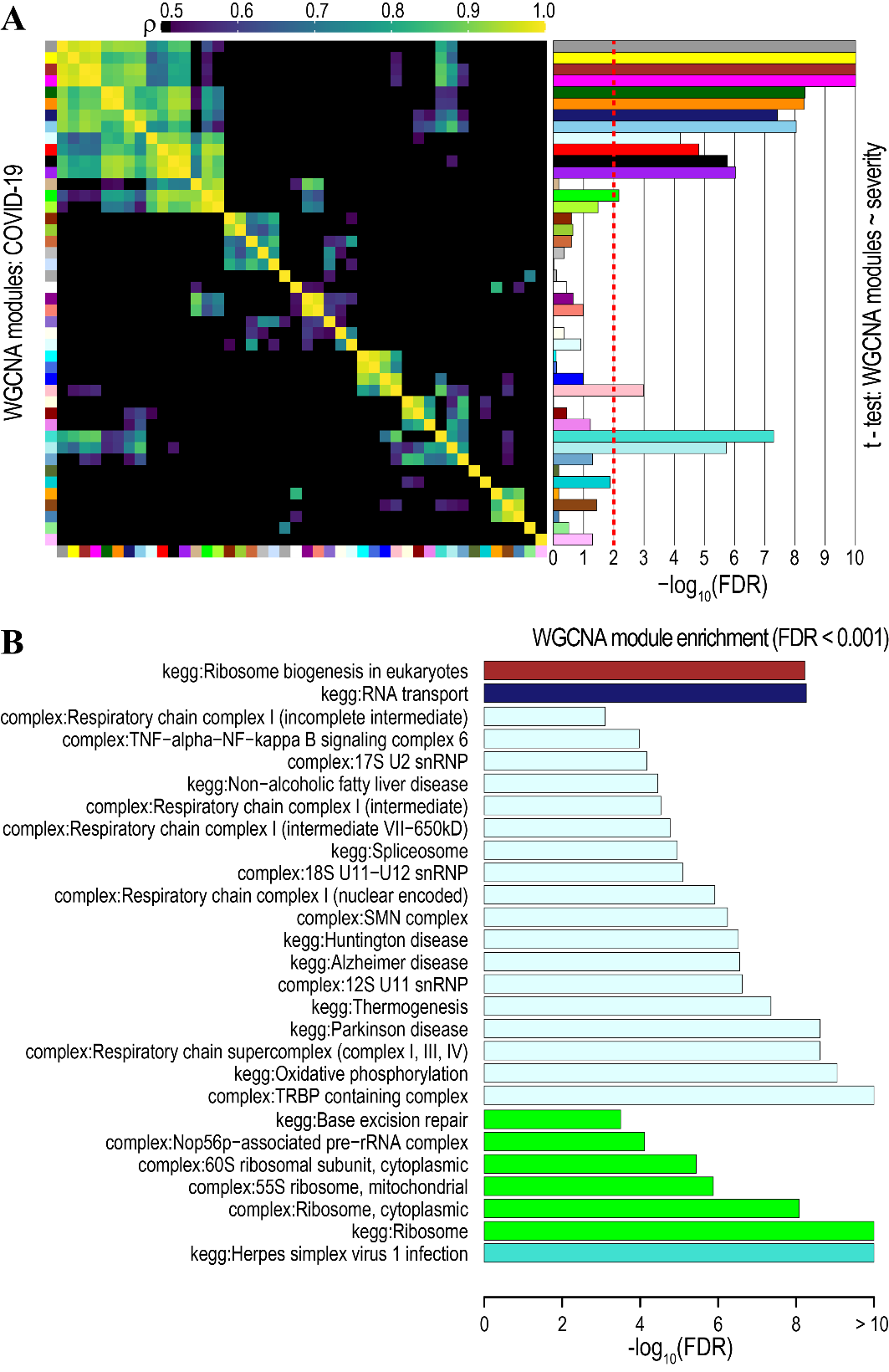


**Figure S11. Association of WGCNA eigengenes (modules) with the severity of the COVID-19 disease.**

**A)** Heatmap plot of the correlations in the eigengene network for COVID-19 patients. The color key for the correlations is shown in the top-left. Eigengenes (modules) are labeled by color and are shown on the left side and the bottom of the heatmap. Barplots on the right demonstrate the significance of WGCNA modules (colored bars) associations with the severity of the COVID-19 disease. -log_10_(FDR) values were derived from the t-tests of WGCNA eigengenes comparing mild and severe COVID-19 cases. The dashed red line indicates a threshold of -log_10_(FDR < 0.01) > 2.

**B)** KEGG/CORUM annotation of WGCNA modules associated significantly (FDR < 0.001) with the severity of the COVID-19 disease.


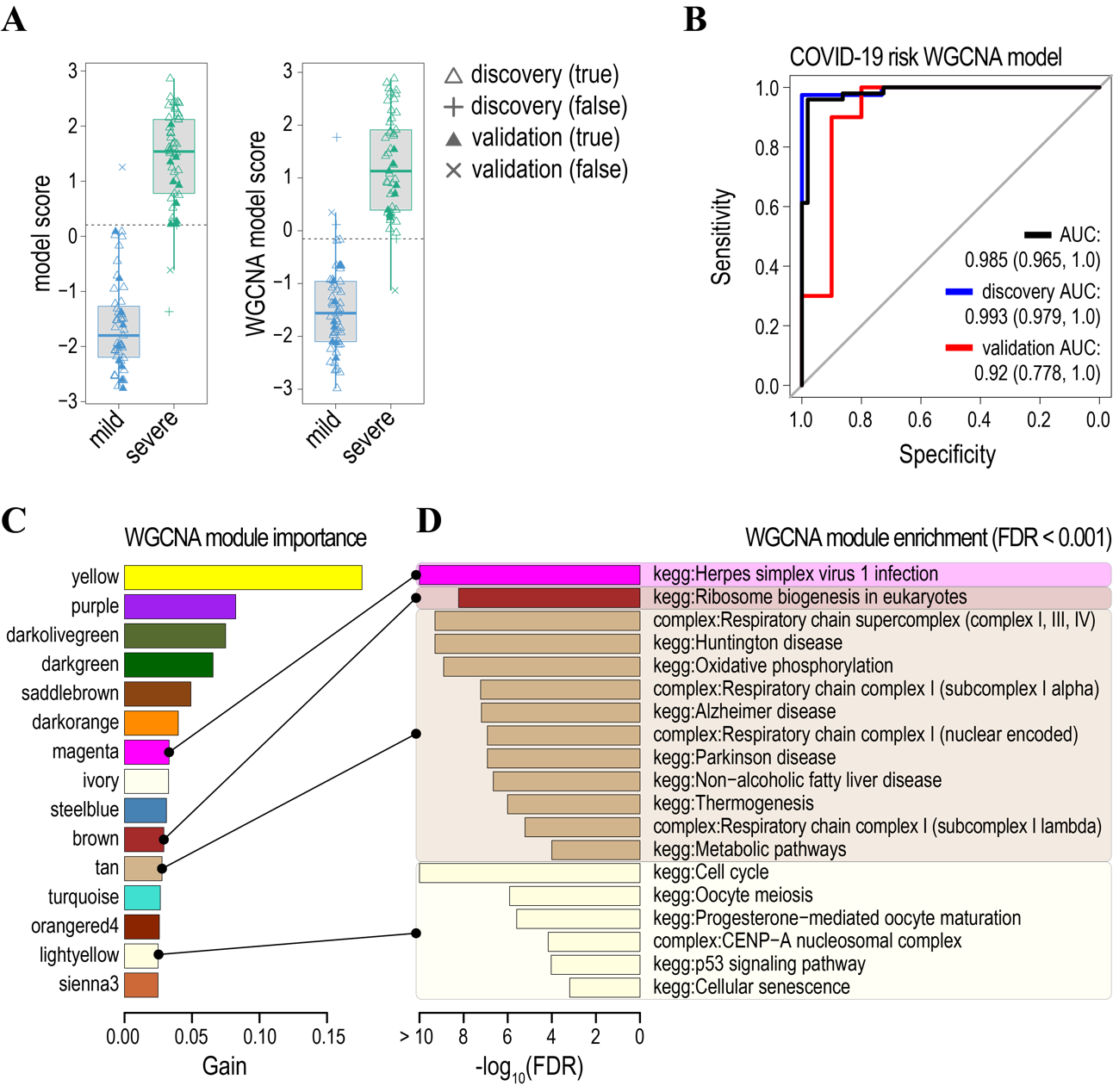


**Figure S12. Annotation of the WGCNA model predicting the severity of the COVID-19 disease.**

**A)** Boxplots of the models’ scores predicting the severity (mild/severe) of the COVID-19 disease in the discovery (open triangles) and validation (filled triangles) cohorts. Models were trained with either gene ensemble noise (left) or WGCNA eigengenes (right) as explanatory variables. Misclassified samples are indicated as (+) for the discovery and (x) for validation cohorts. Samples were randomly partitioned into 80% - discovery and 20% - validation cohorts. Further details of model accuracy are given in Tables 3 and S4.

**B)** ROC curves for the model based on the WGCNA eigengenes. For further details on the WGCNA model accuracy see Tables 2 and S4.

**C)** Ranking of WGCNA eigengenes (modules) according to their importance (Gain).

**D)** KEGG/CORUM annotation of WGCNA modules. The enriched KEGG and CORUM were selected at hypergeometric FDR < 0.01.

**Table S1.** **Kendall correlation of gene ensemble noise with H1N1, sepsis/CAP(CAP) and other sepsis (sepsis) disease states.**

**A) H1N1 (p ≤ 0.05)** $\boldsymbol{\cap}$ **CAP (p ≤ 0.05)** $\boldsymbol{\cap}$ **sepsis (p ≤ 0.05)**

| Gene ensemble (KEGG/CORUM) | H1N1 (τ, p) | CAP (τ, p) | Sepsis (τ, p) | genes |
| --- | --- | --- | --- | --- |
| KEGG: HIF-1 signalling pathway | **τ = 0.373**  **p = 0.0357** | **τ = 0.429**  **p = 0** | **τ = 0.283**  **p = 0** | AKT1; AKT2; ALDOA; ARNT; CAMK2D; CAMK2G; CDKN1A; CDKN1B; CREBBP; CUL2; CYBB; EDN1; EGF; EGLN1; EGLN2; EIF4E2; EIF4EBP1; ELOB; ELOC; ENO1; ENO2; EP300; GAPDH; HIF1A; HK1; HK2; HK3; HMOX1; IFNG; IFNGR1; IFNGR2; IGF1R; IL6R; INSR; LDHA; LTBR; MAP2K1; MAP2K2; MAPK3; MKNK1; MKNK2; NFKB1; NPPA; PDHA1; PDHB; PDK1; PFKFB3; PFKL; PGK1; PIK3CA; PIK3CB; PIK3CD; PIK3R1; PLCG2; PRKCB; RBX1; RPS6; RPS6KB1; RPS6KB2; SLC2A1; STAT3; TFRC; TIMP1; TLR4; VHL |
| KEGG: Peroxisome | **τ = 0.412**  **p = 0.0219** | **τ = 0.323**  **p = 0** | **τ = 0.235**  **p = 0** | ABCD1; ABCD3; ACAA1; ACOT8; ACOX1; ACOX2; ACOX3; ACSL1; ACSL3; ACSL4; ACSL5; ACSL6; AGPS; AMACR; CAT; CRAT; DHRS4; DHRS4L1; ECI2; FAR1; FAR2; GNPAT; GSTK1; HACL1; HMGCL; HSD17B4; IDH1; IDH2; MPV17; MVK; NUDT19; NUDT7; PECR; PEX11B; PEX11G; PEX16; PEX5; PEX6; PEX7; PRDX1; PRDX5; PXMP2; PXMP4; SLC27A2; SOD1; SOD2 |
| KEGG: Necroptosis | **τ = 0.373**  **p = 0.0357** | **τ = 0.309**  **p = 0** | **τ = 0.229**  **p = 0** | AIFM1; BAX; BID; BIRC2; BIRC3; CAMK2D; CAMK2G; CAPN1; CAPN2; CASP1; CASP8; CFLAR; CHMP1A; CHMP1B; CHMP2A; CHMP2B; CHMP3; CHMP4A; CHMP4B; CHMP5; CHMP6; CHMP7; CYBB; CYLD; DNM1L; EIF2AK2; FADD; FAF1; FAS; FASLG; FTH1; FTL; GLUD1; GLUD2; GLUL; H2AFX; H2AFY; H2AFZ; HIST1H2AA; HIST1H2AC; HIST1H2AD; HIST1H2AE; HIST2H2AA3; HIST2H2AA4; HIST2H2AC; HIST3H2A; HMGB1; HSP90AA1; HSP90AB1; IFNAR1; IFNAR2; IFNG; IFNGR1; IFNGR2; IL1A; IL1B; IRF9; JAK1; JAK2; JAK3; JMJD7-PLA2G4B; MLKL; NLRP3; PARP4; PLA2G4A; PLA2G4F; PPID; PYCARD; PYGB; PYGL; RBCK1; RIPK1; RIPK3; RNF31; SHARPIN; SLC25A5; SLC25A6; SMPD1; SPATA2L; SQSTM1; STAT1; STAT2; STAT3; STAT4; STAT5A; STAT5B; STAT6; TICAM1; TLR4; TNF; TNFAIP3; TNFRSF10A; TNFRSF10B; TNFRSF1A; TNFSF10; TRADD; TRAF5; TRPM7; TYK2; USP21; VDAC1; VDAC2; VDAC3; VPS4A; VPS4B; XIAP; ZBP1 |
| KEGG: NOD-like receptor signalling pathway | **τ = 0.501**  **p = 0.0062** | **τ = 0.357**  **p = 0** | **τ = 0.252**  **p = 0** | AIM2; ATG12; ATG5; BCL2L1; BIRC2; BIRC3; BRCC3; CAMP; CARD16; CARD17; CARD6; CARD8; CARD9; CASP1; CASP4; CASP5; CASP8; CCL2; CCL5; CHUK; CTSB; CXCL1; CXCL8; CYBA; CYBB; DEFA1B; DEFA3; DEFA4; DHX33; DNM1L; ERBIN; FADD; GABARAP; GABARAPL1; GABARAPL2; GBP1; GBP2; GBP3; GBP4; GBP5; GSDMD; HSP90AA1; HSP90AB1; IFI16; IFNAR1; IFNAR2; IKBKB; IKBKE; IKBKG; IL18; IL1B; IRAK4; IRF3; IRF7; IRF9; JAK1; JUN; MAP3K7; MAPK13; MAPK14; MAPK3; MAVS; MCU; MEFV; MFN1; MFN2; MYD88; NAIP; NAMPT; NEK7; NFKB1; NFKBIA; NLRC4; NLRP1; NLRP12; NLRP3; NLRP7; NLRX1; NOD2; OAS1; OAS2; OAS3; P2RX7; PLCB1; PLCB2; PRKCD; PSTPIP1; PYCARD; RBCK1; RHOA; RIPK1; RIPK2; RIPK3; RNASEL; RNF31; SHARPIN; STAT1; STAT2; SUGT1; TAB1; TAB2; TAB3; TANK; TBK1; TICAM1; TLR4; TMEM173; TNF; TNFAIP3; TRAF3; TRAF5; TRAF6; TRIP6; TRPM2; TRPM7; TXN; TXN2; TXNIP; TYK2; VDAC1; VDAC2; VDAC3; XIAP |
| KEGG: Fc epsilon RI signalling pathway | **τ = 0.353**  **p = 0.0493** | **τ = 0.342**  **p = 0** | **τ = 0.184**  **p = 0.00007** | AKT1; AKT2; ALOX5; ALOX5AP; BTK; FCER1A; FCER1G; FYN; GAB2; GRB2; INPP5D; JMJD7-PLA2G4B; KRAS; LAT; LCP2; LYN; MAP2K1; MAP2K2; MAP2K3; MAP2K4; MAP2K6; MAP2K7; MAPK13; MAPK14; MAPK3; MS4A2; NRAS; PDPK1; PIK3CA; PIK3CB; PIK3CD; PIK3R1; PLA2G4A; PLA2G4F; PLCG2; RAC2; RAF1; SOS1; SOS2; SYK; TNF; VAV1 |
| KEGG: Autophagy - other | **τ = 0.491**  **p = 0.0067** | **τ = 0.199**  **p = 0.0015** | **τ = 0.137**  **p = 0.0302** | ATG101; ATG12; ATG13; ATG2A; ATG2B; ATG3; ATG4A; ATG4C; ATG5; ATG7; ATG9A; BECN1; GABARAP; GABARAPL1; GABARAPL2; IGBP1; MLST8; PIK3C3; PIK3R4; PPP2CA; PPP2CB; WIPI1; WIPI2 |
| KEGG: Biosynthesis of amino acids | **τ = 0.363**  **p = 0.0455** | **τ = 0.336**  **p = 0** | **τ = 0.274**  **p = 0** | ACO2; ALDH18A1; ALDOA; ALDOC; ARG1; ARG2; ASL; ASNS; BCAT1; BCAT2; CBS; CS; ENO1; ENO2; GAPDH; GLUL; GOT1; GOT2; IDH1; IDH2; IDH3B; IDH3G; MAT2A; MAT2B; MTR; PFKL; PFKM; PFKP; PGAM1; PGK1; PHGDH; PKM; PRPS1; PRPS2; PSPH; PYCR2; PYCR3; RPIA; SDSL; SHMT1; SHMT2; TALDO1; TKT; TKTL1; TPI1 |
| CORUM: TRAPP complex | **τ = 0.412**  **p = 0.0219** | **τ = 0.276**  **p = 0** | **τ = 0.144**  **p = 0.0136** | TRAPPC1; TRAPPC10; TRAPPC11; TRAPPC12; TRAPPC2; TRAPPC2L; TRAPPC3; TRAPPC4; TRAPPC5; TRAPPC6A; TRAPPC6B; TRAPPC8; TRAPPC9 |
| KEGG: Glucagon signalling pathway | **τ = 0.403**  **p = 0.0299** | **τ = 0.211**  **p = 0.0004** | **τ = 0.145**  **p = 0.0122** | ACACA; AKT1; AKT2; ATF4; CALM1; CALM2; CALML4; CAMK2D; CAMK2G; CPT1A; CPT1B; CREB1; CREB3; CREB3L1; CREB5; CREBBP; CRTC2; EP300; FBP1; GNAQ; GNAS; GYS1; LDHA; LDHB; PCK2; PDE3B; PDHA1; PDHB; PFKL; PGAM1; PHKA2; PHKB; PHKG2; PKM; PLCB1; PLCB2; PPP3CA; PPP3CB; PPP3CC; PPP3R1; PPP4C; PPP4R3A; PPP4R3B; PRKAA1; PRKAB1; PRKACB; PRKAG1; PRKAG2; PRMT1; PYGB; PYGL; SIK1; SIK2; SIRT1; SLC2A1 |
| KEGG: Propanoate (propionate) metabolism | **τ = 0.393**  **p = 0.03** | **τ = 0.182**  **p = 0.0081** | **τ = 0.137**  **p = 0.0298** | ABAT; ACACA; ACADM; ACAT1; ACAT2; ACSS1; ACSS2; ALDH6A1; BCKDHA; BCKDHB; DLD; ECHDC1; ECHS1; HADHA; HIBCH; LDHA; LDHB; PCCA; PCCB; SUCLA2; SUCLG1 |
| KEGG: Circadian rhythm | **τ = 0.393**  **p = 0.03** | **τ = 0.353**  **p = 0** | **τ = 0.288**  **p = 0** | ARNTL; BHLHE40; BTRC; CLOCK; CREB1; CRY1; CSNK1D; CUL1; FBXL3; FBXW11; NR1D1; PER1; PRKAA1; PRKAB1; PRKAG1; PRKAG2; RBX1; RORA; SKP1 |
| KEGG: Dopaminergic synapse | **τ = 0.599;**  **p = 0.001005** | **τ = 0.355;**  **p = 0** | **τ = 0.252;**  **p = 0** | AKT1; AKT2; ARNTL; ARRB2; ATF4; ATF6B; CACNA1A; CALM1; CALM2; CALML4; CAMK2D; CAMK2G; CLOCK; CREB1; CREB3; CREB3L1; CREB5; FOS; GNAI2; GNAI3; GNAQ; GNAS; GNB1; GNB2; GNB4; GNB5; GNG10; GNG11; GNG2; GNG7; GNG8; GSK3A; GSK3B; KIF5B; MAOA; MAOB; MAPK13; MAPK14; PLCB1; PLCB2; PPP1CA; PPP1CB; PPP1CC; PPP2CA; PPP2CB; PPP2R2A; PPP2R2B; PPP2R2D; PPP2R3B; PPP2R3C; PPP2R5A; PPP2R5B; PPP2R5C; PPP2R5D; PPP2R5E; PPP3CA; PPP3CB; PPP3CC; PRKACB; PRKCB |
| KEGG: Amyotrophic lateral sclerosis (ALS) | **τ = 0.461**  **p = 0.0102** | **τ = 0.292**  **p = 0** | **τ = 0.189**  **p = 0.00003** | ALS2; BAD; BAX; BCL2L1; BID; CASP1; CASP3; CASP9; CAT; CCS; DAXX; DERL1; GPX1; GRIN1; MAP2K3; MAP2K6; MAP3K5; MAPK13; MAPK14; PPP3CA; PPP3CB; PPP3CC; PPP3R1; RAB5A; SOD1; TNF; TNFRSF1A; TNFRSF1B; TOMM40; TOMM40L |

**B) H1N1 (p ≤ 0.05)** $\boldsymbol{\cap}$ **CAP (p ≤ 0.05)** $\boldsymbol{\cap}$ **sepsis (p > 0.05)**

| **Gene ensemble (KEGG/CORUM)** | **H1N1 (τ, p)** | **CAP (τ, p)** | **Sepsis (τ, p)** | **genes** |
| --- | --- | --- | --- | --- |
| CORUM: Respiratory chain complex I (subcomplex I alpha), mitochondrial | **τ = 0.432**  **p = 0.0178** | **τ = 0.222**  **p = 0.0001** | **τ** = 0.127  p = 0.0929 | NDUFA1; NDUFA10; NDUFA11; NDUFA12; NDUFA13; NDUFA2; NDUFA3; NDUFA5; NDUFA6; NDUFA7; NDUFA8; NDUFA9; NDUFAB1; NDUFB4; NDUFB7; NDUFS1; NDUFS2; NDUFS3; NDUFS6; NDUFS7; NDUFS8; NDUFV1; NDUFV2 |
| CORUM: Respiratory chain complex I (nuclear-encoded subunits), mitochondrial | **τ = 0.383**  **p = 0.0323** | **τ = 0.169**  **p = 0.0271** | **τ** = 0.092  p = 1 | NDUFA1; NDUFA10; NDUFA11; NDUFA12; NDUFA13; NDUFA2; NDUFA3; NDUFA4; NDUFA5; NDUFA6; NDUFA7; NDUFA8; NDUFA9; NDUFAB1; NDUFB1; NDUFB10; NDUFB11; NDUFB2; NDUFB3; NDUFB4; NDUFB5; NDUFB6; NDUFB7; NDUFB8; NDUFB9; NDUFC1; NDUFC2; NDUFS1; NDUFS2; NDUFS3; NDUFS4; NDUFS5; NDUFS6; NDUFS7; NDUFS8; NDUFV1; NDUFV2 |
| KEGG: Osteoclast differentiation | **τ = 0.373**  **p = 0.0357** | **τ = 0.279**  **p = 0** | **τ** = 0.13  p = 0.066 | ACP5; AKT1; AKT2; BLNK; BTK; CHUK; CREB1; CSF1R; CTSK; CYBA; CYLD; FCGR1A; FCGR2A; FCGR2B; FCGR2C; FCGR3A; FCGR3B; FHL2; FOS; FOSL2; FYN; GAB2; GRB2; IFNAR1; IFNAR2; IFNG; IFNGR1; IFNGR2; IKBKB; IKBKG; IL1A; IL1B; IL1R1; IRF9; ITGB3; JAK1; JUN; JUNB; JUND; LCK; LCP2; LILRA1; LILRA2; LILRA4; LILRA5; LILRA6; LILRB1; LILRB2; LILRB3; LILRB4; MAP2K1; MAP2K6; MAP2K7; MAP3K14; MAP3K7; MAPK13; MAPK14; MAPK3; MITF; NCF1; NCF2; NCF4; NFATC1; NFKB1; NFKB2; NFKBIA; OSCAR; PIK3CA; PIK3CB; PIK3CD; PIK3R1; PLCG2; PPARG; PPP3CA; PPP3CB; PPP3CC; PPP3R1; RELB; SIRPA; SIRPB1; SIRPG; SOCS1; SOCS3; SPI1; SQSTM1; STAT1; STAT2; SYK; TAB1; TAB2; TGFB1; TGFBR2; TNF; TNFRSF1A; TRAF6; TYK2; TYROBP |
| KEGG: Tight junction | **τ = 0.412**  **p = 0.0219** | **τ = 0.208**  **p = 0.0006** | **τ** = 0.127  p = 0.0933 | ACTB; ACTG1; ACTN1; ACTN4; ACTR2; ACTR3; ARHGAP17; ARHGEF18; ARHGEF2; CD1C; CDC42; CDK4; CLDN15; CLDN5; CLDN9; DLG1; EPB41L4B; EZR; F11R; HCLS1; HSPA4; ITGB1; JUN; LLGL2; MAP2K7; MAP3K1; MAP3K5; MICALL2; MSN; MYH9; MYL12A; MYL12B; MYL6; MYL6B; MYL9; NEDD4; NEDD4L; OCLN; PARD3; PARD6A; PCNA; PPP2CA; PPP2CB; PPP2R2A; PPP2R2B; PPP2R2D; PRKAA1; PRKAB1; PRKACB; PRKAG1; PRKAG2; PRKCE; PRKCI; PRKCZ; RAB13; RAB8A; RAB8B; RAP1A; RAP2C; RAPGEF2; RAPGEF6; RHOA; ROCK1; ROCK2; RUNX1; SCRIB; SLC9A3R1; STK11; TIAM1; TJP2; TUBA1A; TUBA1B; TUBA1C; TUBA4A; TUBA8; VASP; WASL; WHAMM; YBX3 |
| KEGG: Axon guidance | **τ = 0.461**  **p = 0.0102** | **τ = 0.165**  **p = 0.0423** | **τ** = 0.074  p = 1 | ABLIM1; ARHGEF12; BMPR2; CAMK2D; CAMK2G; CDC42; CDK5; CFL1; CXCR4; EFNA1; EFNB3; EPHA4; EPHB1; EPHB4; FES; FYN; GNAI2; GNAI3; GSK3B; ILK; ITGB1; KRAS; LIMK2; LRRC4; MAPK3; MYL12A; MYL12B; MYL5; MYL9; NCK1; NCK2; NFATC3; NRAS; NTNG2; PAK1; PAK2; PAK3; PAK4; PARD3; PARD6A; PDK1; PIK3CA; PIK3CB; PIK3CD; PIK3R1; PLCG2; PLXNA2; PLXNA3; PLXNB2; PLXNC1; PPP3CA; PPP3CB; PPP3CC; PPP3R1; PRKCZ; PTPN11; RAC2; RAF1; RASA1; RHOA; ROBO3; ROCK1; ROCK2; RRAS; RYK; SEMA4A; SEMA4B; SEMA4C; SRGAP2; SRGAP3; SSH1; SSH2; SSH3 |

**Table S2. Confusion tables for the models predicting mortality/survivorship of sepsis patients based on the estimates of gene ensemble noise and WGCNA eigengenes.**

**A) All sepsis (sepsis/CAP and other sepsis) patients**

|  | **discovery** | | **Actual** | |  | **validation** | | **Actual** | |
| --- | --- | --- | --- | --- | --- | --- | --- | --- | --- |
|  |  |  | **survived** | **deceased** |  |  |  | **survived** | **deceased** |
| **Gene Ensemble Noise** | **predicted** | **survived** | **164**  **(84.5%)** | 17  (24.6%) |  | **predicted** | **survived** | **137**  **(80.1%)** | 18  (40.0%) |
|  |  | **deceased** | 30  (15.5%) | **52**  **(75.4%)** |  |  | **deceased** | 34  (19.9%) | **27**  **(60.0%)** |
|  |  | |  | |  |  | |  | |
|  | **discovery** | | **Actual** | |  | **validation** | | **Actual** | |
|  |  |  | **survived** | **deceased** |  |  |  | **survived** | **deceased** |
| **WGCNA** | **predicted** | **survived** | **155**  **(79.9%)** | 13  (18.8%) |  | **predicted** | **survived** | **105**  **(61.4%)** | 9  (20.0%) |
|  |  | **deceased** | 39  (20.1%) | **56**  **(81.2%)** |  |  | **deceased** | 66  (38.6%) | **36**  **(80.0%)** |

**B) sepsis/CAP patients**

|  | **discovery** | | **Actual** | |  | **validation** | | **Actual** | |
| --- | --- | --- | --- | --- | --- | --- | --- | --- | --- |
|  |  |  | **survived** | **deceased** |  |  |  | **survived** | **deceased** |
| **Gene Ensemble Noise** | **predicted** | **survived** | **58**  **(72.5%)** | 3  (12.0%) |  | **predicted** | **survived** | **46**  **(73.0%)** | 2  (13.3%) |
|  |  | **deceased** | 22  (27.5%) | **22**  **(88.0%)** |  |  | **deceased** | 17  (27.0%) | **13**  **(86.7%)** |
|  |  | |  | |  |  | |  | |
|  | **discovery** | | **Actual** | |  | **validation** | | **Actual** | |
|  |  |  | **survived** | **deceased** |  |  |  | **survived** | **deceased** |
| **WGCNA** | **predicted** | **survived** | **64**  **(80.0%)** | 5  (20.0%) |  | **predicted** | **survived** | **47**  **(74.6%)** | 2  (13.3%) |
|  |  | **deceased** | 16  (20.0%) | **20**  **(80.0%)** |  |  | **deceased** | 16  (25.4%) | **13**  **(86.7%)** |

**C) Other sepsis patients**

|  | **discovery** | | **Actual** | |  | **validation** | | **Actual** | |
| --- | --- | --- | --- | --- | --- | --- | --- | --- | --- |
|  |  |  | **survived** | **deceased** |  |  |  | **survived** | **deceased** |
| **Gene Ensemble Noise** | **predicted** | **survived** | **92**  **(80.7%)** | 11  (25.0%) |  | **predicted** | **survived** | **78**  **(72.2%)** | 6  (20.0%) |
|  |  | **deceased** | 22  (19.3%) | **33**  **(75.0%)** |  |  | **deceased** | 30  (27.8%) | **24**  **(80.0%)** |
|  |  | |  | |  |  | |  | |
|  | **discovery** | | **Actual** | |  | **validation** | | **Actual** | |
|  |  |  | **survived** | **deceased** |  |  |  | **survived** | **deceased** |
| **WGCNA** | **predicted** | **survived** | **91**  **(79.8%)** | 7  (15.9%) |  | **predicted** | **survived** | **83**  **(76.9%)** | 9  (30.0%) |
|  |  | **deceased** | 23  (20.2%) | **37**  **(84.1%)** |  |  | **deceased** | 25  (23.1%) | **21**  **(70.0%)** |

**Table S3. Relative contribution (gain %, cover %, frequency %) of gene ensemble noise features to the models predicting mortality/survivorship of sepsis (sepsis/CAP and other sepsis) patients**

| **Gene ensemble (KEGG/CORUM)** | **Gain; Cover; Frequency** | | | **genes** |
| --- | --- | --- | --- | --- |
|  | **All sepsis** | **Sepsis/ CAP** | **Other sepsis** |  |
| CORUM: 18S U11-U12 snRNP | **14.537;**  **10.6;**  **8.054** |  | **5.818;**  **5.966;**  **5.376** | DHX15; PDCD7; PRPF8; RNPC3; SF3B1; SF3B2; SF3B3; SF3B4; SF3B5; SF3B6; SNRNP25; SNRNP35; SNRNP48; SNRPB; SNRPD1; SNRPD2; SNRPD3; SNRPE; SNRPF; SNRPG; YBX1; ZCRB1; ZMAT5; ZRSR2 |
| CORUM: LARC complex (LCR-associated remodelling complex) | **8.069;**  **7.116;**  **6.04** | **1.444;**  **3.508;**  **6.061** |  | ACTB; ACTL6A; ARID1A; CHD4; DPF2; GATAD2B; HDAC1; MBD2; MBD3; RBBP4; SMARCA4; SMARCC2; SMARCD2; SMARCE1 |
| KEGG: Primary immunodeficiency | **5.845;**  **6.663;**  **6.711** |  | **4.555;**  **5.175;**  **4.301** | ADA; BLNK; BTK; CD19; CD3D; CD3E; CD40; CD79A; CD8A; CD8B; CIITA; DCLRE1C; IKBKG; IL2RG; IL7R; JAK3; LCK; ORAI1; PTPRC; RFX5; RFXANK; TAP1; TAP2; TNFRSF13B |
| KEGG: Legionellosis | **2.575;**  **1.944;**  **2.013** | **9.579;**  **5.673;**  **3.03** |  | ARF1; BCL2L13; BNIP3; C3; CASP1; CASP3; CASP7; CASP8; CASP9; CD14; CLK1; CR1; CXCL1; CXCL8; EEF1G; HBS1L; HSF1; HSPA1A; HSPA1B; HSPA1L; HSPA6; HSPA8; HSPD1; IL18; IL1B; ITGAM; ITGB2; MYD88; NAIP; NFKB1; NFKB2; NFKBIA; NLRC4; PYCARD; RAB1A; RAB1B; SAR1A; SAR1B; SEC22B; TLR2; TLR4; TLR5; TNF; VCP |
| KEGG: Epithelial cell signalling in Helicobacter pylori infection | **2.472;**  **4.05;**  **3.356** |  | **4.263;**  **3.426;**  **5.376** | ADAM10; ADAM17; ATP6AP1; ATP6V0A2; ATP6V0B; ATP6V0C; ATP6V0D1; ATP6V0E1; ATP6V0E2; ATP6V1A; ATP6V1B2; ATP6V1C1; ATP6V1D; ATP6V1E1; ATP6V1E2; ATP6V1F; ATP6V1G1; ATP6V1H; CASP3; CCL5; CDC42; CHUK; CSK; CXCL1; CXCL8; CXCR1; CXCR2; F11R; HBEGF; IKBKB; IKBKG; JUN; LYN; MAP2K4; MAP3K14; MAPK13; MAPK14; NFKB1; NFKBIA; PAK1; PLCG2; PTPN11; TCIRG1 |
| KEGG: Endocrine resistance | **2.208;**  **2.692;**  **4.027** |  |  | ADCY3; ADCY4; ADCY7; AKT1; AKT2; ARAF; BAD; BAX; BIK; BRAF; CARM1; CDK4; CDKN1A; CDKN1B; CDKN2A; CDKN2C; E2F1; E2F3; FOS; GNAS; GPER1; GRB2; HBEGF; IGF1R; JAG2; JUN; KRAS; MAP2K1; MAP2K2; MAPK13; MAPK14; MAPK3; MED1; MMP9; NCOR1; NOTCH2; NRAS; PIK3CA; PIK3CB; PIK3CD; PIK3R1; PRKACB; RAF1; RB1; RPS6KB1; RPS6KB2; SHC1; SOS1; SOS2; SP1 |
| KEGG: Rheumatoid arthritis | **1.96;**  **2.399;**  **2.685** |  | **8.817;**  **8.538;**  **5.376** | ACP5; ATP6AP1; ATP6V0A2; ATP6V0B; ATP6V0C; ATP6V0D1; ATP6V0E1; ATP6V0E2; ATP6V1A; ATP6V1B2; ATP6V1C1; ATP6V1D; ATP6V1E1; ATP6V1E2; ATP6V1F; ATP6V1G1; ATP6V1H; CCL2; CCL20; CCL3; CCL3L1; CCL5; CD86; CTSK; CTSL; CXCL1; CXCL5; CXCL8; FOS; HLA-DMA; HLA-DMB; HLA-DOB; HLA-DPA1; HLA-DPB1; HLA-DQA1; HLA-DQB1; HLA-DRA; HLA-DRB1; HLA-DRB5; ICAM1; IFNG; IL15; IL18; IL1A; IL1B; ITGAL; ITGB2; JUN; LTB; MMP1; TCIRG1; TGFB1; TLR2; TLR4; TNF; TNFSF13; TNFSF13B |
| KEGG: Purine metabolism | **1.404;**  **1.705;**  **2.685** |  |  | ADA; ADA2; ADCY3; ADCY4; ADCY7; ADPRM; ADSL; ADSS; AK1; AK2; AK3; AK4; AMPD2; AMPD3; APRT; ATIC; CANT1; DCK; DGUOK; ENPP4; ENTPD1; ENTPD4; ENTPD5; ENTPD6; FHIT; GART; GMPR; GMPR2; GMPS; GUCY1A1; GUCY1B1; GUCY2D; GUK1; HDDC3; HPRT1; IMPDH1; IMPDH2; ITPA; NME1; NME1-NME2; NME3; NME4; NME6; NT5C; NT5C2; NT5C3A; NT5M; NTPCR; NUDT16; NUDT5; NUDT9; PAICS; PAPSS1; PAPSS2; PDE1B; PDE2A; PDE3B; PDE4A; PDE4B; PDE4D; PDE6D; PDE6G; PDE7A; PDE8A; PGM1; PGM2; PKM; PNP; PPAT; PRPS1; PRPS2; PRUNE1; RRM1; RRM2; RRM2B |
| KEGG: Salivary secretion | **0.979;**  **1.44;**  **2.013** | **5.848;**  **3.685;**  **6.061** | **0.548;**  **1.377;**  **2.151** | ADCY3; ADCY4; ADCY7; ADRB2; AMY1A; AQP5; ATP1A1; ATP1B1; ATP1B3; ATP2B1; ATP2B4; BST1; CALM1; CALM2; CALML4; CAMP; CD38; CST2; CST3; CST4; GNAQ; GNAS; GUCY1A1; GUCY1B1; KCNMA1; KCNN4; LYZ; PLCB1; PLCB2; PRH1; PRKACB; PRKCB; SLC9A1 |
| KEGG: Progesterone-mediated oocyte maturation | **0.739;**  **0.379;**  **0.671** |  |  | ADCY3; ADCY4; ADCY7; AKT1; AKT2; ANAPC1; ANAPC11; ANAPC13; ANAPC4; ANAPC5; ARAF; AURKA; BRAF; BUB1; CCNA1; CCNA2; CCNB1; CCNB2; CDC16; CDC25B; CDC26; CDC27; CDK1; CDK2; CPEB2; CPEB3; CPEB4; GNAI2; GNAI3; HSP90AA1; HSP90AB1; IGF1R; KIF22; KRAS; MAD1L1; MAD2L1; MAD2L2; MAP2K1; MAPK13; MAPK14; MAPK3; PDE3B; PIK3CA; PIK3CB; PIK3CD; PIK3R1; PKMYT1; PRKACB; RAF1; RPS6KA1; RPS6KA2; RPS6KA3; SPDYE1; STK10 |
| CORUM: 12S U11 snRNP | **0.68;**  **1.354;**  **0.671** |  | **5.814;**  **4.818;**  **5.376** | PDCD7; PRKRIP1; SNRNP25; SNRNP35; SNRNP48; SNRPB; SNRPD1; SNRPD2; SNRPD3; SNRPE; SNRPF; SNRPG; SRSF7; YBX1 |
| KEGG: Cytosolic DNA-sensing pathway | **0.621;**  **0.525;**  **1.342** |  | **7.675;**  **5.934;**  **4.301** | ADAR; AIM2; CASP1; CCL4; CCL4L1; CCL5; CGAS; CHUK; CXCL10; DDX58; IKBKB; IKBKE; IKBKG; IL18; IL1B; IRF3; IRF7; MAVS; NFKB1; NFKBIA; POLR1C; POLR1D; POLR2E; POLR2F; POLR2H; POLR2K; POLR2L; POLR3C; POLR3D; POLR3E; POLR3GL; POLR3K; PYCARD; RIPK1; RIPK3; TBK1; TMEM173; TREX1; ZBP1 |
| KEGG: Glutathione metabolism | **0.326;**  **0.323;**  **0.671** |  |  | ANPEP; CHAC2; G6PD; GCLC; GCLM; GGT1; GPX1; GPX4; GPX7; GSR; GSS; GSTK1; GSTM3; GSTO1; GSTP1; HPGDS; IDH1; IDH2; LAP3; MGST1; MGST2; MGST3; NAT8B; ODC1; OPLAH; PGD; RRM1; RRM2; RRM2B; SMS; SRM; TXNDC12 |
| KEGG: Steroid biosynthesis |  | **9.044;**  **9.84;**  **9.091** |  | CYP2R1; CYP51A1; DHCR24; DHCR7; EBP; FDFT1; LIPA; MSMO1; NSDHL; SOAT1; SQLE; TM7SF2 |
| KEGG: RNA polymerase |  | **6.459;**  **3.452;**  **6.061** |  | POLR1B; POLR1C; POLR1D; POLR1E; POLR2A; POLR2B; POLR2C; POLR2E; POLR2F; POLR2G; POLR2H; POLR2I; POLR2J; POLR2J3; POLR2K; POLR2L; POLR3C; POLR3D; POLR3E; POLR3GL; POLR3K; ZNRD1 |
| KEGG: mTOR signalling pathway |  | **5.646;**  **5.038;**  **3.03** |  | AKT1; AKT1S1; AKT2; ATP6V1A; ATP6V1B2; ATP6V1C1; ATP6V1D; ATP6V1E1; ATP6V1E2; ATP6V1F; ATP6V1G1; ATP6V1H; BRAF; CAB39; CASTOR1; CHUK; CLIP1; DDIT4; DVL1; EIF4B; EIF4E2; EIF4EBP1; FLCN; FNIP2; FZD1; FZD2; GRB10; GRB2; GSK3B; IGF1R; IKBKB; INSR; KRAS; LAMTOR1; LAMTOR2; LAMTOR3; LAMTOR4; MAP2K1; MAP2K2; MAPK3; MAPKAP1; MIOS; MLST8; NPRL2; NPRL3; NRAS; PDPK1; PIK3CA; PIK3CB; PIK3CD; PIK3R1; PRKAA1; PRKCB; PRR5; PTEN; RAF1; RHEB; RHOA; RICTOR; RPS6; RPS6KA1; RPS6KA2; RPS6KA3; RPS6KB1; RPS6KB2; RRAGA; RRAGB; RRAGC; RRAGD; SEC13; SESN2; SGK1; SLC38A9; SLC3A2; SOS1; SOS2; STK11; STRADA; STRADB; TBC1D7; TELO2; TNF; TNFRSF1A; TSC2; TTI1; ULK1; WDR59; WNT3 |
| CORUM: SMN complex |  | **3.084;**  **2.097;**  **3.03** |  | DDX20; GEMIN4; GEMIN7; GEMIN8; SNRPB; SNRPD1; SNRPD2; SNRPD3; SNRPE; SNRPF; SNRPG; STRAP |
| KEGG: Ribosome biogenesis in eukaryotes |  | **1.874;**  **3.782;**  **3.03** |  | BMS1; CSNK2A1; CSNK2A2; CSNK2B; DKC1; DROSHA; EIF6; EMG1; FBL; FCF1; GNL2; GNL3; GNL3L; GTPBP4; HEATR1; IMP3; IMP4; LSG1; MPHOSPH10; NHP2; NOB1; NOP10; NOP56; NOP58; NXF1; NXT1; NXT2; POP4; POP5; POP7; PWP2; RAN; RCL1; REXO1; REXO2; RPP25L; RPP38; RRP7A; SBDS; SNU13; TCOF1; UTP14A; UTP14C; UTP18; UTP4; UTP6; WDR36; WDR75; XPO1; XRN1; XRN2 |
| KEGG: Prion diseases |  | **0.571;**  **2.383;**  **3.03** |  | BAX; C1QA; C1QB; C1QC; C5; CCL5; EGR1; ELK1; FYN; HSPA1A; HSPA5; IL1A; IL1B; LAMC1; MAP2K1; MAP2K2; MAPK3; PRKACB; PRNP; SOD1; STIP1 |
| CORUM: Kinase maturation complex 1 |  | **0.228;**  **2.612;**  **3.03** |  | CDC37; HSP90AA1; HSP90AB1; HSPA4; MAP2K5; MAP3K3; PDRG1; PFDN2; TRAF7; YWHAB; YWHAG; YWHAH; YWHAQ; YWHAZ |
| KEGG: Arachidonic acid metabolism |  |  | **8.65;**  **10.792;**  **11.828** | AKR1C3; ALOX12; ALOX5; CBR1; CYP4A22; CYP4F2; CYP4F3; GGT1; GPX1; GPX7; HPGDS; JMJD7-PLA2G4B; LTA4H; LTC4S; PLA2G12A; PLA2G4A; PLA2G4F; PLB1; PRXL2B; PTGDS; PTGES; PTGES3; PTGS1; PTGS2; TBXAS1 |
| KEGG: Collecting duct acid secretion |  |  | **5.466;**  **7.8;**  **8.602** | ATP6V0A2; ATP6V0C; ATP6V0D1; ATP6V0E1; ATP6V0E2; ATP6V1A; ATP6V1B2; ATP6V1C1; ATP6V1D; ATP6V1E1; ATP6V1E2; ATP6V1F; ATP6V1G1; CA2; SLC4A1; TCIRG1 |
| KEGG: Regulation of lipolysis in adipocytes |  |  | **5.229;**  **3.744;**  **5.376** | ABHD5; ADCY3; ADCY4; ADCY7; ADRB2; AKT1; AKT2; GNAI2; GNAI3; GNAS; INSR; IRS2; MGLL; NPPA; PDE3B; PIK3CA; PIK3CB; PIK3CD; PIK3R1; PRKACB; PTGS1; PTGS2 |
| KEGG: Leishmaniasis |  |  | **4.508;**  **6.629;**  **6.452** | C3; CR1; CYBA; CYBB; ELK1; FCGR1A; FCGR2A; FCGR2C; FCGR3A; FCGR3B; FOS; HLA-DMA; HLA-DMB; HLA-DOB; HLA-DPA1; HLA-DPB1; HLA-DQA1; HLA-DQB1; HLA-DRA; HLA-DRB1; HLA-DRB5; IFNG; IFNGR1; IFNGR2; IL10; IL1A; IL1B; IRAK1; IRAK4; ITGA4; ITGAM; ITGB1; ITGB2; JAK1; JAK2; JUN; MAP3K7; MAPK13; MAPK14; MAPK3; MARCKSL1; MYD88; NCF1; NCF2; NCF4; NFKB1; NFKBIA; PRKCB; PTGS2; PTPN6; STAT1; TAB1; TAB2; TGFB1; TLR2; TLR4; TNF; TRAF6 |

**Table S4. Confusion tables for the models predicting the severity of the COVID-19 disease based on the estimates of gene ensemble noise and WGCNA eigengenes**

|  | **discovery** | | **Actual** | |  | **validation** | | **Actual** | |
| --- | --- | --- | --- | --- | --- | --- | --- | --- | --- |
|  |  |  | **mild** | **severe** |  |  |  | **survived** | **severe** |
| **Gene Ensemble Noise** | **predicted** | **mild** | **39**  **(95.1%)** | 1  (2.6%) |  | **predicted** | **survived** | **9**  **(90.0%)** | 0  (0%) |
|  |  | **severe** | 2  (4.9%) | **38**  **(97.4%)** |  |  | **deceased** | 1  (10.0%) | **10**  **(100%)** |
|  |  | |  | |  |  | |  | |
|  | **discovery** | | **Actual** | |  | **validation** | | **Actual** | |
|  |  |  | **survived** | **severe** |  |  |  | **survived** | **severe** |
| **WGCNA** | **predicted** | **mild** | **39**  **(95.1%)** | 1  (2.6%) |  | **predicted** | **mild** | **9**  **(90.0%)** | 1  (10.0%) |
|  |  | **severe** | 2  (4.9%) | **38**  **(97.4%)** |  |  | **severe** | 1  (10.0%) | **9**  **(90.0%)** |

**Table S5. Relative contribution (gain %, cover %, frequency %) of gene ensemble noise features to the model predicting the severity of the COVID-19 disease**

| **Gene ensemble (KEGG/CORUM)** | **Gain** | **Cover** | **Frequency** | **genes** |
| --- | --- | --- | --- | --- |
| CORUM: TLE1 corepressor complex (MASH1 promoter-corepressor complex) | 25.154 | 11.151 | 8.434 | ACTB; HSPA4; MYH10; NCL; NONO; NPM1; PARP1; RAD50; TLE1; TOP2B |
| CORUM: CtBP complex | 10.964 | 9.911 | 9.639 | CBX4; CDYL; CTBP1; CTBP2; EHMT1; EHMT2; HDAC1; HDAC2; KDM1A; LCOR; RCOR1; RCOR3; RREB1; ZEB1; ZEB2; ZNF217; ZNF516 |
| CORUM: Cytochrome c oxidase, mitochondrial | 10.816 | 14.23 | 12.048 | COX4I1; COX5A; COX5B; COX6A2; COX6B1; COX6C; COX7A1; COX7B; COX7C; COX8B; MT-CO1; MT-CO2; MT-CO3 |
| CORUM: Glycosphingolipid biosynthesis - lacto and neolacto series | 9.491 | 7.768 | 7.229 | A4GALT; ABO; B3GALNT1; B3GALT1; B3GALT2; B3GALT5; B3GNT2; B3GNT3; B3GNT4; B3GNT5; B4GALT1; B4GALT2; B4GALT3; B4GALT4; FUT1; FUT2; FUT3; FUT4; FUT5; FUT6; FUT7; FUT9; GCNT2; ST3GAL3; ST3GAL4; ST3GAL6; ST8SIA1 |
| CORUM: TNF-alpha-NF-kappa B signaling complex 10 | 7.842 | 7.066 | 6.024 | ATG16L1; CCAR1; CDC37; CHUK; HSP90AA1; HSP90AB1; IKBKG; NFKBIB; TBK1; TXLNA |
| CORUM: Starch and sucrose metabolism | 5.75 | 5.075 | 6.024 | AGL; AMY1A; AMY1B; AMY1C; AMY2A; AMY2B; ENPP1; ENPP3; G6PC; G6PC2; G6PC3; GAA; GANC; GBA3; GBE1; GCK; GPI; GYG1; GYG2; GYS1; GYS2; HK1; HK2; HK3; HKDC1; MGAM; MGAM2; PGM1; PGM2; PGM2L1; PYGB; PYGL; PYGM; SI; TREH; UGP2 |
| KEGG: Nitrogen metabolism | 4.019 | 4.176 | 4.819 | CA1; CA12; CA13; CA14; CA2; CA3; CA4; CA5A; CA5B; CA6; CA7; CA8; CA9; CPS1; GLUD1; GLUD2; GLUL |
| KEGG: RNA polymerase | 3.628 | 5.855 | 6.024 | POLR1A; POLR1B; POLR1C; POLR1D; POLR1E; POLR2A; POLR2B; POLR2C; POLR2D; POLR2E; POLR2F; POLR2G; POLR2H; POLR2I; POLR2J; POLR2J2; POLR2J3; POLR2K; POLR2L; POLR3A; POLR3B; POLR3C; POLR3D; POLR3E; POLR3F; POLR3G; POLR3GL; POLR3H; POLR3K; TWISTNB; ZNRD1 |
| CORUM: TRBP containing complex | 3.339 | 3.378 | 3.614 | DICER1; EIF6; MOV10; RPL10A; RPL11; RPL12; RPL13; RPL14; RPL15; RPL18A; RPL19; RPL21; RPL27; RPL28; RPL30; RPL32; RPL34; RPL35A; RPL36; RPL37A; RPL7; RPL7A; RPLP1; RPLP2; RSL24D1 |
| KEGG: Other glycan degradation | 3.225 | 2.529 | 3.614 | AGA; ENGASE; FUCA1; FUCA2; GBA; GBA2; GLB1; HEXA; HEXB; HEXD; MAN2B1; MAN2B2; MAN2C1; MANBA; NEU1; NEU2; NEU3; NEU4 |
| CORUM: Fanconi anemia FAAP100 complex | 2.873 | 4.227 | 3.614 | FAAP100; FAAP24; FANCA; FANCB; FANCC; FANCE; FANCF; FANCG; FANCL; FANCM |
| KEGG: JAK-STAT signaling pathway | 2.624 | 2.825 | 2.41 | AKT1; AKT2; AKT3; AOX1; BCL2; BCL2L1; CCND1; CCND2; CCND3; CDKN1A; CISH; CNTF; CNTFR; CREBBP; CRLF2; CSF2; CSF2RA; CSF2RB; CSF3; CSF3R; CSH1; CSH2; CTF1; EGF; EGFR; EP300; EPO; EPOR; FHL1; GFAP; GH1; GH2; GHR; GRB2; HRAS; IFNA1; IFNA10; IFNA13; IFNA14; IFNA16; IFNA17; IFNA2; IFNA21; IFNA4; IFNA5; IFNA6; IFNA7; IFNA8; IFNAR1; IFNAR2; IFNB1; IFNE; IFNG; IFNGR1; IFNGR2; IFNK; IFNL1; IFNL2; IFNL3; IFNLR1; IFNW1; IL10; IL10RA; IL10RB; IL11; IL11RA; IL12A; IL12B; IL12RB1; IL12RB2; IL13; IL13RA1; IL13RA2; IL15; IL15RA; IL17D; IL19; IL2; IL20; IL20RA; IL20RB; IL21; IL21R; IL22; IL22RA1; IL22RA2; IL23A; IL23R; IL24; IL27RA; IL2RA; IL2RB; IL2RG; IL3; IL3RA; IL4; IL4R; IL5; IL5RA; IL6; IL6R; IL6ST; IL7; IL7R; IL9; IL9R; IRF9; JAK1; JAK2; JAK3; LEP; LEPR; LIF; LIFR; MCL1; MPL; MTOR; MYC; OSM; OSMR; PDGFA; PDGFB; PDGFRA; PDGFRB; PIAS1; PIAS2; PIAS3; PIAS4; PIK3CA; PIK3CB; PIK3CD; PIK3R1; PIK3R2; PIK3R3; PIM1; PRL; PRLR; PTPN11; PTPN2; PTPN6; RAF1; SOCS1; SOCS2; SOCS3; SOCS4; SOCS5; SOCS6; SOCS7; SOS1; SOS2; STAM; STAM2; STAT1; STAT2; STAT3; STAT4; STAT5A; STAT5B; STAT6; THPO; TSLP; TYK2 |
| KEGG: Antigen processing and presentation | 2.492 | 7.233 | 9.639 | B2M; CALR; CANX; CD4; CD74; CD8A; CD8B; CIITA; CREB1; CTSB; CTSL; CTSS; HLA-A; HLA-B; HLA-C; HLA-DMA; HLA-DMB; HLA-DOA; HLA-DOB; HLA-DPA1; HLA-DPB1; HLA-DQA1; HLA-DQA2; HLA-DQB1; HLA-DRA; HLA-DRB1; HLA-DRB5; HLA-E; HLA-F; HLA-G; HSP90AA1; HSP90AB1; HSPA1A; HSPA1B; HSPA1L; HSPA2; HSPA4; HSPA5; HSPA6; HSPA8; IFI30; IFNG; KIR2DL1; KIR2DL3; KIR2DL4; KIR2DS4; KIR3DL1; KIR3DL2; KIR3DL3; KLRC1; KLRC2; KLRC3; KLRC4; KLRD1; LGMN; NFYA; NFYB; NFYC; PDIA3; PSME1; PSME2; PSME3; RFX5; RFXANK; RFXAP; TAP1; TAP2; TAPBP; TNF |
| KEGG: Rap1 signaling pathway | 2.118 | 5.469 | 6.024 | ACTB; ACTG1; ADCY1; ADCY2; ADCY3; ADCY4; ADCY5; ADCY6; ADCY7; ADCY8; ADCY9; ADORA2A; ADORA2B; AFDN; AKT1; AKT2; AKT3; ANGPT1; ANGPT2; ANGPT4; APBB1IP; ARAP3; BCAR1; BRAF; CALM1; CALM2; CALM3; CALML3; CALML4; CALML5; CALML6; CDC42; CDH1; CNR1; CRK; CRKL; CSF1; CSF1R; CTNNB1; CTNND1; DOCK4; DRD2; EFNA1; EFNA2; EFNA3; EFNA4; EFNA5; EGF; EGFR; EPHA2; F2R; F2RL3; FARP2; FGF1; FGF10; FGF16; FGF17; FGF18; FGF19; FGF2; FGF20; FGF21; FGF22; FGF23; FGF3; FGF4; FGF5; FGF6; FGF7; FGF8; FGF9; FGFR1; FGFR2; FGFR3; FGFR4; FLT1; FLT4; FPR1; FYB1; GNAI1; GNAI2; GNAI3; GNAO1; GNAQ; GNAS; GRIN1; GRIN2A; GRIN2B; HGF; HRAS; ID1; IGF1; IGF1R; INS; INSR; ITGA2B; ITGAL; ITGAM; ITGB1; ITGB2; ITGB3; KDR; KIT; KITLG; KRAS; KRIT1; LAT; LCP2; LPAR1; LPAR2; LPAR3; LPAR4; LPAR5; MAGI1; MAGI2; MAGI3; MAP2K1; MAP2K2; MAP2K3; MAP2K6; MAPK1; MAPK11; MAPK12; MAPK13; MAPK14; MAPK3; MET; MRAS; NGF; NGFR; NRAS; P2RY1; PARD3; PARD6A; PARD6B; PARD6G; PDGFA; PDGFB; PDGFC; PDGFD; PDGFRA; PDGFRB; PFN1; PFN2; PFN3; PFN4; PGF; PIK3CA; PIK3CB; PIK3CD; PIK3R1; PIK3R2; PIK3R3; PLCB1; PLCB2; PLCB3; PLCB4; PLCE1; PLCG1; PRKCA; PRKCB; PRKCG; PRKCI; PRKCZ; PRKD1; PRKD2; PRKD3; RAC1; RAC2; RAC3; RAF1; RALA; RALB; RALGDS; RAP1A; RAP1B; RAP1GAP; RAPGEF1; RAPGEF2; RAPGEF3; RAPGEF4; RAPGEF5; RAPGEF6; RASGRP2; RASGRP3; RASSF5; RGS14; RHOA; RRAS; SIPA1; SIPA1L1; SIPA1L2; SIPA1L3; SKAP1; SRC; TEK; THBS1; TIAM1; TLN1; TLN2; VASP; VAV2; VEGFA; VEGFB; VEGFC; VEGFD |
| complex:Respiratory chain complex I (subcomplex I alpha), mitochondrial | 1.919 | 1.472 | 2.41 | MT-ND6; NDUFA1; NDUFA10; NDUFA11; NDUFA12; NDUFA13; NDUFA2; NDUFA3; NDUFA5; NDUFA6; NDUFA7; NDUFA8; NDUFA9; NDUFAB1; NDUFB4; NDUFB7; NDUFS1; NDUFS2; NDUFS3; NDUFS6; NDUFS7; NDUFS8; NDUFV1; NDUFV2; NDUFV3 |
| KEGG: Ubiquinone and other terpenoid-quinone biosynthesis | 1.526 | 3.493 | 3.614 | COQ2; COQ3; COQ5; COQ6; COQ7; GGCX; HPD; NQO1; TAT; VKORC1; VKORC1L1 |
| CORUM: TNF-alpha-NF-kappa B signaling complex 6 | 1.502 | 2.512 | 2.41 | CDC37; CHUK; FBL; HSP90AA1; HSP90AB1; IKBKB; IKBKG; MAP3K14; RPL30; RPL4; RPL6; RPL8; RPS11; RPS13 |
| KEGG: Glutathione metabolism | 0.718 | 1.632 | 2.41 | ANPEP; CHAC1; CHAC2; G6PD; GCLC; GCLM; GGCT; GGT1; GGT5; GGT6; GGT7; GPX1; GPX2; GPX3; GPX4; GPX5; GPX6; GPX7; GPX8; GSR; GSS; GSTA1; GSTA2; GSTA3; GSTA4; GSTA5; GSTK1; GSTM1; GSTM2; GSTM3; GSTM4; GSTM5; GSTO1; GSTO2; GSTP1; GSTT2; GSTT2B; HPGDS; IDH1; IDH2; LAP3; MGST1; MGST2; MGST3; NAT8; NAT8B; ODC1; OPLAH; PGD; RRM1; RRM2; RRM2B; SMS; SRM; TXNDC12 |
